# Mode of birth and risk of inflammatory bowel disease in offspring: an updated systematic review and meta-analysis

**DOI:** 10.3389/frph.2026.1776110

**Published:** 2026-04-08

**Authors:** Hana Taha, Mohammad Al-Shalalfeh, Almothana Khalil, Yumna Khasawneh, Aseel Abu Ata, Hala AbuEin, Ahmad Issa, Taher Alhawamdeh, Mishkah BaniMustafa, Mohammed Qussay Al-Sabbagh, Linus Jönsson

**Affiliations:** 1 Department of Family and Community Medicine, School of Medicine, The University of Jordan, Amman, Jordan; 2 Department of Neurobiology, Care Sciences and Society, Karolinska Institutet, Solna, Sweden; 3 King Khalid Hospital, Kharj, Saudi Arabia; 4 Department of Neurology, University of Kansas Medical Center, Kansas, KS, United States

**Keywords:** caesarean delivery, Crohn's disease, early-life, inflammatory bowel disease, meta-analysis, ulcerative colitis

## Abstract

**Background:**

Cesarean section (CS) rates continue rising worldwide, raising concerns about long-term offspring health consequences, including inflammatory bowel disease (IBD). This systematic review and meta-analysis evaluate the association between CS and risk of IBD, Crohn's disease (CD), and ulcerative colitis (UC).

**Methods:**

PubMed, Scopus, CENTRAL, and Web of Science were searched through June 2025. Eligible studies included observational cohorts and case-control studies reporting CS vs. vaginal delivery (VD) and IBD outcomes. Data extraction and risk of bias assessment were performed independently. Pooled relative risks (RRs), hazard ratios (HRs), and odds ratios (ORs) were calculated using fixed or random-effects models. Subgroup analyses and publication bias assessment were conducted.

**Results:**

Twenty-two studies comprising over 13 million births were included. Unadjusted analyses showed no association between CS and IBD (RR: 0.98, 95% CI: 0.88–1.08), CD (RR: 0.99, 95% CI: 0.88–1.12), however, an inverse association was observed for UC (RR: 0.82, 95% CI: 0.72–0.95). Regional variation was observed, with CS associated with reduced IBD risk in Denmark, Switzerland, and Norway, but increased risk in Germany and Australia. Adjusted analyses consistently demonstrated no association: IBD (HR: 1.14, 95% CI: 0.99–1.30; OR: 0.91, 95% CI: 0.65–1.25), CD (HR: 1.07, 95% CI: 0.90–1.28; OR: 1.11, 95% CI: 0.98–1.26), and UC (HR: 0.96, 95% CI: 0.87–1.05; OR: 1.05, 95% CI: 0.86–1.27). No publication bias was detected.

**Conclusion:**

Across over 13 million births, delivery mode was not associated with IBD, CD, or UC risk. Despite biologically plausible mechanisms linking CS to altered microbiome patterns, epidemiological evidence does not support CS as an independent IBD risk factor. These findings provide reassurance for clinical counseling regarding CS and long-term IBD risk.

**Systematic Review Registration:**

https://www.crd.york.ac.uk/PROSPERO/view/CRD420251237413, PROSPERO CRD420251237413.

## 1 Introduction

The global rate of cesarean section (CS) deliveries has risen sharply over recent decades, now accounting for roughly 20 percent of all births worldwide (1–5). While CS is essential in many obstetric situations, increasing attention has focused on its potential long-term consequences for offspring health (6). Although commonly perceived as a safer option for newborns, accumulating evidence suggests that children delivered via CS may be at higher risk of respiratory problems (7), infections (8), obesity (9), asthma (7), and immune-related disorders (10).

One proposed mechanism linking CS to these outcomes is its impact on early-life microbial colonization (11). Vaginal birth exposes infants to maternal vaginal and gut microbiota, while CS delivery leads to colonization dominated by skin-associated microbes. These early differences may alter immune system development (12, 13). Disruptions in early microbial establishment may impair immune regulation and contribute to immune-mediated conditions, including inflammatory bowel disease (IBD), later in life (12, 14).

IBD, encompassing Crohn's disease (CD) and ulcerative colitis (UC), is characterized by chronic, dysregulated inflammation of the gastrointestinal tract (15). Although genetic susceptibility plays a role, early environmental exposures have become increasingly recognized as important contributors to disease risk (15). The simultaneous rise in CS rates and IBD incidence, combined with evolving perinatal practices, has intensified interest in whether birth mode may influence long-term immune development and subsequent disease risk (15).

Despite biologically plausible mechanisms, epidemiologic findings remain inconsistent. Large cohort studies have produced conflicting results: a Scottish national birth cohort reported no association between CS and pediatric onset of IBD (16), whereas a Danish nationwide cohort observed a significantly increased risk among children delivered by CS (17). These discrepancies may reflect differences in study populations, exposure definitions, and analytic methods. Importantly, previous meta-analyses were limited by the inability to differentiate between elective and emergency CS and by insufficient adjustment for key confounders such as parental IBD and age at IBD onset (18, 19). Without addressing these limitations, it remains unclear whether observed associations are causal or confounded.

These gaps highlight the need for an updated and methodologically robust synthesis of the available evidence. In this systematic review and meta-analysis, we aim to evaluate the association between CS and the onset of IBD in offspring using the latest and most rigorous data. By distinguishing elective from emergency CS, adjusting for maternal IBD, and conducting subgroup and sensitivity analyses to explore geographic and methodological heterogeneity, this review provides a comprehensive assessment of current evidence. The findings have important implications for clinical counseling and public health decision-making in the context of rising global CS rates.

## 2 Methods

### 2.1 Protocol and registration

This systematic review was conducted following the Preferred Reporting Items for Systematic Reviews and Meta-Analyses (PRISMA) statement and registered in the International Prospective Register of Systematic Reviews (PROSPERO CRD420251237413).

### 2.2 Search strategy

A comprehensive literature search was performed in PubMed, Scopus, CENTRAL, and Web of Science from inception to June 16, 2025. The search combined controlled vocabulary and free-text terms related to IBD, UC, CD, and mode of delivery (Supplementary Table S1). To enhance specificity, we applied filters restricting results to English-language, human studies and excluded review articles. The initial search yielded 800 records. We also manually screened the reference lists of included studies to identify additional eligible publications.

### 2.3 Study selection

Three reviewers independently screened titles and abstracts in Rayyan (20), with blinding enabled. Full texts of potentially relevant studies were then assessed by three other reviewers using predefined eligibility criteria. Studies were eligible if they reported the number of patients with IBD, UC, or CD and provided data on mode of delivery [e.g., CS or vaginal delivery (VD)] in both affected individuals and controls. Studies were required to present effect estimates [unadjusted or adjusted odds ratios (ORs), relative risks (RRs), or hazard ratios (HRs)] or sufficient raw data to calculate these measures. Eligible study designs included cohort, case-control, and randomized controlled trials, and both pediatric and adult populations were considered. Only English-language full-text articles were included.

Studies were excluded if they did not report the number of IBD, UC, or CD cases; did not describe the mode of delivery; or lacked effect estimates with insufficient data for calculation. We also excluded conference abstracts, review articles, animal studies, case reports, inappropriate designs, and studies focused solely on experimental or interventional outcomes unrelated to delivery mode. Discrepancies at any stage were resolved by consensus.

### 2.4 Data extraction

The data were extracted independently by four reviewers using a standardized Excel form. Extracted variables included study ID, location, design, outcome definitions, sample size, number of IBD, UC, and CD cases, and details on elective and emergency CS when available. Reported effect estimates (ORs, RRs, HRs) for overall IBD and for UC and CD separately were recorded. Information on potential confounders, including maternal IBD and age, was also collected when reported. Disagreements were resolved by consensus; unresolved issues were adjudicated by a fifth reviewer, whose decision was final.

### 2.5 Quality assessment

Four reviewers independently assessed study quality using the Newcastle-Ottawa Scale (NOS) (21). The NOS evaluates non-randomized studies across three domains: selection, comparability, and exposure or outcome (for case-control and cohort studies, respectively). The scale includes eight items, with a maximum score of 9. Scores of 0–3 indicate low quality, 4–6 moderate quality, and 7–9 high quality (Lo et al., 2014). A follow-up rate above 80% was considered adequate. Discrepancies were settled by consensus.

### 2.6 Statistical analysis

The association between CS and disease risk was analyzed using both adjusted and unadjusted measures. Separate meta-analyses were conducted for overall IBD, CD, and UC. The IBD analysis included all studies reporting composite IBD outcomes, regardless of subtype specification, whereas the CD and UC analyses included only studies reporting these specific diagnoses. Therefore, some study populations overlapped across analyses. For raw data counts, we calculated the pooled RR with 95% confidence interval (CI). For studies that provided pre-adjusted estimates, we pooled multivariate-adjusted HR and OR, each with a 95% CI. Statistical heterogeneity was assessed using multiple complementary measures, including the I^2^ statistic, Cochran's *Q* test, *τ*^2^, and H^2^. The I^2^ statistic quantified the proportion of total variability attributable to between study heterogeneity, with values of 0%–40% considered low, 30%–60% moderate, 50%–90% substantial, and 75%–100% considerable heterogeneity. Cochran's *Q* test evaluated the null hypothesis of a common underlying effect, with a *p* value < 0.10 indicating statistically significant heterogeneity. The between study variance was estimated using *τ*^2^, and H^2^ was calculated to express the ratio of total variability to within study variability. Substantial heterogeneity was defined as I^2^ > 50% or a Q test *p* value < 0.10. A leave-one-out sensitivity analysis was conducted to assess the robustness of the results. Publication bias was evaluated using funnel plots and Egger's regression test. Funnel plots are scatter plots of study effect sizes against their precision (measured by standard error). In the absence of publication bias, studies should be distributed symmetrically around the pooled effect estimate, forming an inverted funnel shape smaller studies (with larger standard errors) show more scatter at the bottom, while larger studies (with smaller standard errors) cluster near the top. Asymmetry suggests potential publication bias, where small studies with non-significant or unfavorable results may be missing from the literature. Egger's test provides a statistical assessment of funnel plot asymmetry, with *p* < 0.05 indicating significant asymmetry suggestive of publication bias. All described statistical analyses were performed using Stata statistical software version 17.

## 3 Results

### 3.1 Literature search and study selection

**Figure 1:**
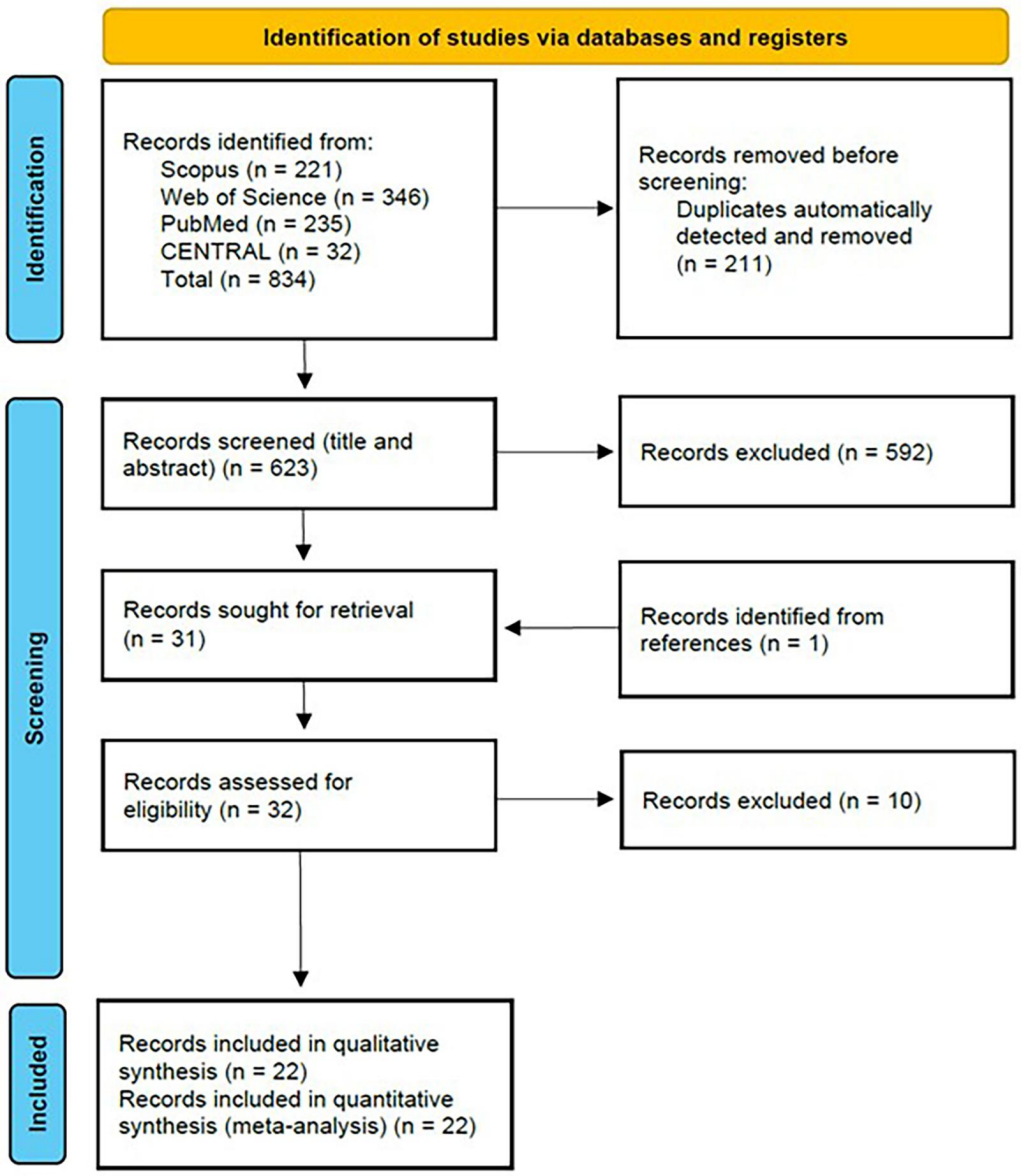
PRISMA flow diagram illustrates the systematic literature search and study selection process.

The initial systematic search identified 834 potentially relevant articles. Following the removal of 211 duplicate records, 623 unique articles underwent abstract screening. Application of the predetermined exclusion criteria resulted in the elimination of 592 articles. Subsequently, 31 articles identified through database searching and one additional article identified through reference list examination proceeded to full-text review. After a comprehensive full-text assessment, 10 records were excluded based on eligibility criteria, yielding a final cohort of 22 articles for inclusion in the systematic review and meta-analysis (Figure 1, Supplementary Table S2**).**

### 3.2 Characteristics of included studies

The systematic review encompassed 22 studies published between 2007 and 2023, comprising 6 case-control studies and 16 retrospective cohort studies. Notably, Burnett et al., 2020 (22) conducted parallel analyses using both clinical and administrative cohorts, reporting effect estimates separately for each dataset. The geographical distribution demonstrated regional diversity, with 11 studies originating from Central and Northern Europe, 5 from North America, 3 from the United Kingdom, 2 from the Middle East, and 1 from Australia. Most included studies identified inf IBD cases through International Classification of Diseases (ICD) coding systems, while the remainder utilized clinical diagnosis or self-reported diagnoses. Collectively, the included studies encompassed 2,264,652 CS deliveries and 11,459,742 VD. A comprehensive summary of individual study characteristics is presented in Table 1.

**Table 1:** Individual characteristics of the included studies.

Study ID	Location	Population	Study design	IBD identification method	CS	VD	Females *n* (%)
Ponsonby et al. 2009 (23)	Australia	All live births in Victoria from 1983 to 1998	Retrospective cohort	Clinical diagnosis was confirmed by histology or radiology	177,871	820,728	485,165 (48.6%)
Zamstein et al. 2022 (24)	Israel	infants born in Soroka University Medical Center from 1991 to 2014, excluding instrumental vaginal deliveries	Retrospective cohort	ICD-9 codes	6,376	961	3,883 (52.9%)
Andersen et al. 2020 (25)	Denmark	All live births in Denmark from 1973 to 2016	Retrospective cohort	ICD 8 and 10 codes	400,795	2,271,913	-
Soullane et al. 2021 (26)	Canada	All children born in Quebec hospitals between 2006 and 2019	Retrospective cohort	ICD-10 code	248,963	685,910	460,025 (49.2%)
Decker et al. 2010 (27)	Germany	Children and young adolescents visiting outpatient clinics at 42 hospitals in germany	Case control	Clinical diagnosis and histological confirmation	226	1,060	629 (48.9%)
kristensen et al. 2016 (28)	Denmark	All children born in Denmark from 1997 to 2012	Retrospective cohort	ICD-10 code	124,130	666,439	-
Bager et al. 2012 (17)	Denmark	All children born in Denmark from 1973 to 2008	Retrospective cohort	ICD-8 from 1977 to 1993 and ICD-10 since 1994	288,784	1,809,255	-
Malmborg et al. 2012 (29)	Sweden	Children born in Sweden from 1973 to 2006 who were diagnosed with Crohn's disease before age 16	Case control	ICD-8, ICD-9 and ICD-10 codes	2,036	14,939	7,147 (42.1%)
Bernstein et al. 2016 (30)	Canada	Individuals born in Manitoba (1970–2010)	Retrospective cohort	Records from the University of Manitoba IBD Epidemiology Database (UMIBDED)	1,420	10,739	6,010 (49.4%)
Hellsing et al. 2022 (11)	Sweden	full-term individuals born in Sweden (1990–2000), followed through 2017	Retrospective cohort	ICD-9 and ICD-10 codes	127,337	975,131	536,407 (48.7%)
Davidesko et al. 2022 (31)	Israel	Offspring from singleton, uncomplicated deliveries (1991–2014) at a tertiary medical center, followed to age 18	Retrospective cohort	ICD-9 codes	13,242	125,990	69,583 (50%)
Roberts et al. 2011 (15)	England, UK	Children and young adults born between 1970 and 1989 in a defined region of South East England	Retrospective cohort	ICD-8, ICD-9 and ICD-10 codes	18,025	223,793	-
Räisänen et al. 2021 (32)	Finland	Participants of Finnish Health in Teens (Fin-HIT) born between 2000 and 2005	Retrospective cohort	ICD-10 code	1,812	8,857	-
Black et al. 2015 (33)	Scotland, UK	All term (≥37 completed weeks of gestation) singleton live births in first-time mothers between 1993 and 2007 in Scotland	Retrospective cohort	Scottish Morbidity Records 01 (SMR01)	68,370	252,917	156,894 (48.8%)
Spangmose et al. 2023 (34)	Denmark	All individuals born in Denmark from 1994 to 2014	Retrospective cohort	ICD-8 and ICD-10 codes	253,203	1,095,500	656,736 (48.7%)
Burgess et al. 2022 (16)	Scotland, UK	All children born in Scotland throughout 1981 to 2017 within the Scottish Morbidity Record maternity inpatient dataset [SMR02]	Retrospective cohort	ICD-9 and ICD-10 codes	404,653	1,609,198	-
Hutfless et al. 2012 (35)	California, US	All children (aged 17 and under) who were “Kaiser Permanente Northern California” system members for at least one consecutive year between 1996 and 2006	Case control	ICD-9-CM codes	608	2,661	1,647 (51.2%)
Sonntag et al. 2007 (36)	Germany	Patients with Crohn's Disease (CD), Ulcerative Colitis (UC), and healthy controls; recruited nationally and from University Hospital of Muenster	Case control	Clinical diagnosis and histological assessment when available	180	2,970	1,569 (57.3%)
Bengtson et al. 2010 (37)	Norway	Individuals born between 1967 and 2015 in 4 countries of southeastern Norway	Retrospective cohort	Norwegian Medical Birth Registry	56,579	642,044	339,033 (48.5%)
Kuenzig et al. 2020 (38)	Canada	Children born in Ontario between 2006 and 2015	Case-control	ICD-10 codes	512	1,336	792 (42.9%)
Burnett et al, 2020 (22)	Canada	Children born in Nova Scotia between 1988 and 2014 (clinical cohort) and 1989–1993 (administrative cohort), followed up to 2014	Retrospective cohort	Clinical cohort: Records in the Nova Scotia Atlee Perinatal Database (NSAPD) Administrative cohort: ICD-9 and ICD-10CA	61,248 8,156	201,457 34,750	127,501 (48.5%) 21,208 (49.4%)
Lautenschlager et al. 2020 (39)	Switzerland	Adults with IBD enrolled in the Swiss IBD Cohort Study (SIBDCS); matched with childhood friends as controls	Case-control	Self-reported diagnosis	126	1,194	826 (56.5%)

### 3.3 Quality assessment

Quality appraisal of the 6 case-control studies revealed that 3 studies achieved high-quality ratings, while 3 were classified as moderate quality. The moderate-quality studies demonstrated methodological limitations primarily related to case definition, representativeness of cases, and control selection procedures (Table 2). Among the 16 cohort studies, all attained high-quality ratings according to established assessment criteria. The predominant methodological concern identified across cohort studies pertained to comparability between cohorts, with secondary limitations noted in outcome assessment methodology and adequacy of follow-up duration (Table 3)**.**

**Table 2:** Quality assessment of included case-control studies.

	Selection				Comparability	Exposure			
Study	Is the case definition adequate	Representativeness of the cases	Selection of controls	Definition of controls	Comparability of Cases and Controlson the Basis of the Design or Analysis #	Ascertainment of exposure	Same methods of ascertainment for cases and controls	Non-response rate	Total score
Decker et al. 2010 (27)	*	*	-	*	*	-	*	-	5
Malmborg et al. 2012 (29)	*	*	*	*	**	*	*	*	9
Hutfless et al. 2012 (35)	*	*	*	-	**	*	*	*	8
Sonntag et al. 2007 (36)	-	*	*	*	-	-	*	*	5
Kuenzig et al. 2020 (38)	-	*	*	*	**	*	*	*	8
Lautenschlager et al. 2020 (39)	-	-	*	*	*	-	*	-	4

Each “*” = 1 point (star) awarded for meeting that specific methodological criterion.

**Table 3:** Quality assessment of included cohort studies.

	Selection				Comparability	Outcome			
Study	Representativeness of the exposed cohort	Selection of the non exposed cohort	Ascertainment of exposure	Demonstration that outcome of interest was not present at start of study	Comparability of cohorts on the basis of the design or analysis	Assessment of outcome	Was follow-up long enough for outcomes to occur	Adequacy of follow up	Total score
Ponsonby et al. 2009 (23)	*	*	*	*	**	*	*	*	9
Zamstein et al. 2002 (24)	*	*	*	*	**	*	*	*	9
Andersen et al. 2020 (25)	*	*	*	*	**	*	*	*	9
Soullane et al. 2021 (26)	*	*	*	*	**	*	*	*	9
kristensen et al. 2016 (28)	*	*	*	*	*	-	*	*	7
Bager et al. 2012 (17)	*	*	*	*	**	-	*	*	8
Bernstein et al. 2016 (30)	*	*	*	*	*	*	*	*	8
Hellsing et al. 2022 (11)	*	*	*	*	**	*	*	*	9
Davidesko et al. 2022 (31)	*	*	*	*	**	*	*	*	9
Roberts et al. 2011 (15)	*	*	*	*	*	*	*	*	8
Räisänen et al. 2021 (32)	*	*	*	*	*	*	*	-	7
Black et al. 2015 (33)	*	*	*	*	*	*	*	-	7
Spangmose et al. 2023 (34)	*	*	*	*	**	*	*	*	9
Burgess et al. 2022 (16)	*	*	*	*	**	*	*	*	9
Bengtson et al. 2010 (37)	*	*	*	*	-	*	*	*	7
Burnett et al. 2020 (22)	*	*	*	*	*	*	*	*	8

Each “*” = 1 point (star) awarded for meeting that specific methodological criterion.

### 3.4 Meta-Analysis of unadjusted risk ratios

#### 3.4.1 Inflammatory bowel disease

**Figure 2:**
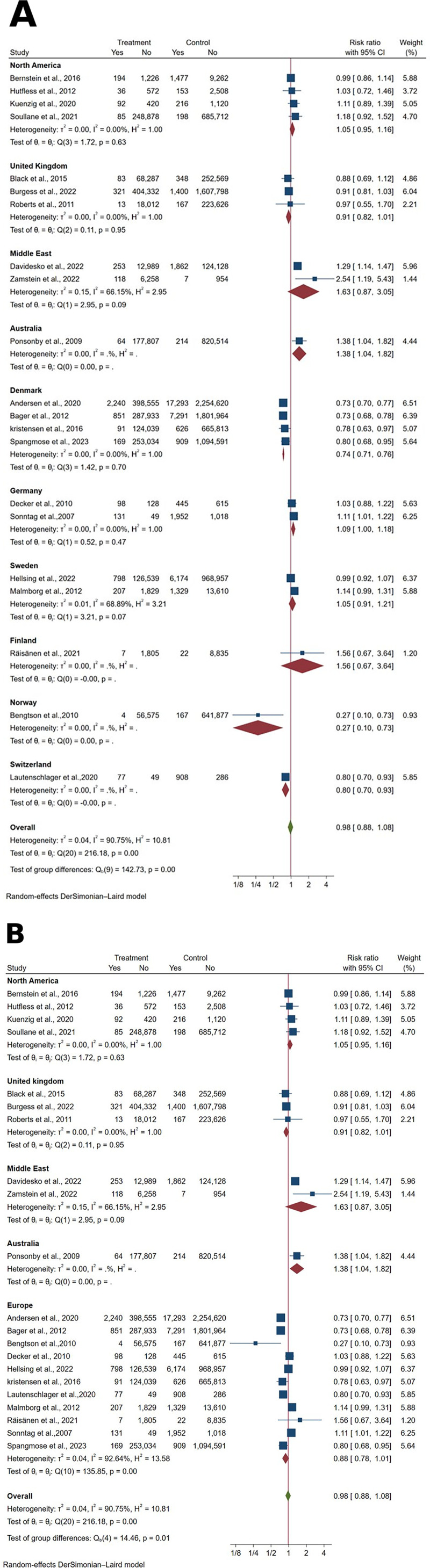
**(A)** forest plot of unadjusted risk ratios for inflammatory bowel disease development comparing cesarean section vs. vaginal delivery, stratified by geographic region. Treatment group represents cesarean section deliveries; Control group represents vaginal deliveries. Risk ratio/hazard ratio/odds ratio <1.0 favors CS (lower IBD/CD/UC risk), while values >1.0 favor VD (lower risk). Each horizontal line represents one study's 95% confidence interval (CI), with the point estimate shown by a square marker (size proportional to study weight). The vertical line at 1.0 represents no association. The diamond represents the pooled effect estimate and 95% CI across all studies. I^2^, tau^2^, H^2^, and Cochran's *Q* test assess between-study heterogeneity (see Methods for interpretation). **(B)** Forest plot of unadjusted risk ratios for inflammatory bowel disease development comparing cesarean section vs. vaginal delivery, with all European countries combined into a single analytical group. Treatment group represents cesarean section deliveries; Control group represents vaginal deliveries. Risk ratio/hazard ratio/odds ratio <1.0 favors CS (lower IBD/CD/UC risk), while values >1.0 favor VD (lower risk). Each horizontal line represents one study's 95% confidence interval (CI), with the point estimate shown by a square marker (size proportional to study weight). The vertical line at 1.0 represents no association. The diamond represents the pooled effect estimate and 95% CI across all studies. I^2^, tau^2^, H^2^, and Cochran's *Q* test assess between-study heterogeneity (see Methods for interpretation).

Twenty-one articles reported IBD case counts, encompassing 11,223,535 participants delivered vaginally and 2,195,248 delivered by cesarean section. The pooled analysis revealed no statistically significant association between mode of delivery and IBD development risk [RR: 0.98, 95% CI: (0.88–1.08), *p* = 0.64] (Figure 2A). Substantial heterogeneity was observed among the included studies (I^2^ = 90.66%, *p* < 0.001).

Geographic stratification revealed significant regional variation in the relationship between cesarean delivery and IBD risk. In Denmark, cesarean section was associated with a 26% reduction in IBD risk [RR: 0.74, 95% CI: (0.71–0.76), *p* < 0.001]. Similarly, Switzerland demonstrated a 20% risk reduction [RR: 0.80, 95% CI: (0.70–0.93), *p* < 0.001], and Norway exhibited a substantial 73% risk reduction [RR: 0.27, 95% CI: (0.10–0.73), *p* = 0.01]. Conversely, cesarean delivery in Germany was associated with a 9% increased IBD risk [RR: 1.09, 95% CI: (1.00–1.18), *p* = 0.04], while Australia demonstrated a 38% increased risk [RR: 1.38, 95% CI: (1.04–1.82), *p* = 0.02]. The test for subgroup differences achieved statistical significance (*p* < 0.001).

When European countries were combined into a single analytical group, cesarean section demonstrated a non-significant 12% reduction in IBD risk [RR: 0.88, 95% CI: (0.78–1.01), *p* = 0.07] (Figure 2B). The test for subgroup differences remained statistically significant (*p* = 0.006).

#### 3.4.2 Crohn's disease

**Figure 3:**
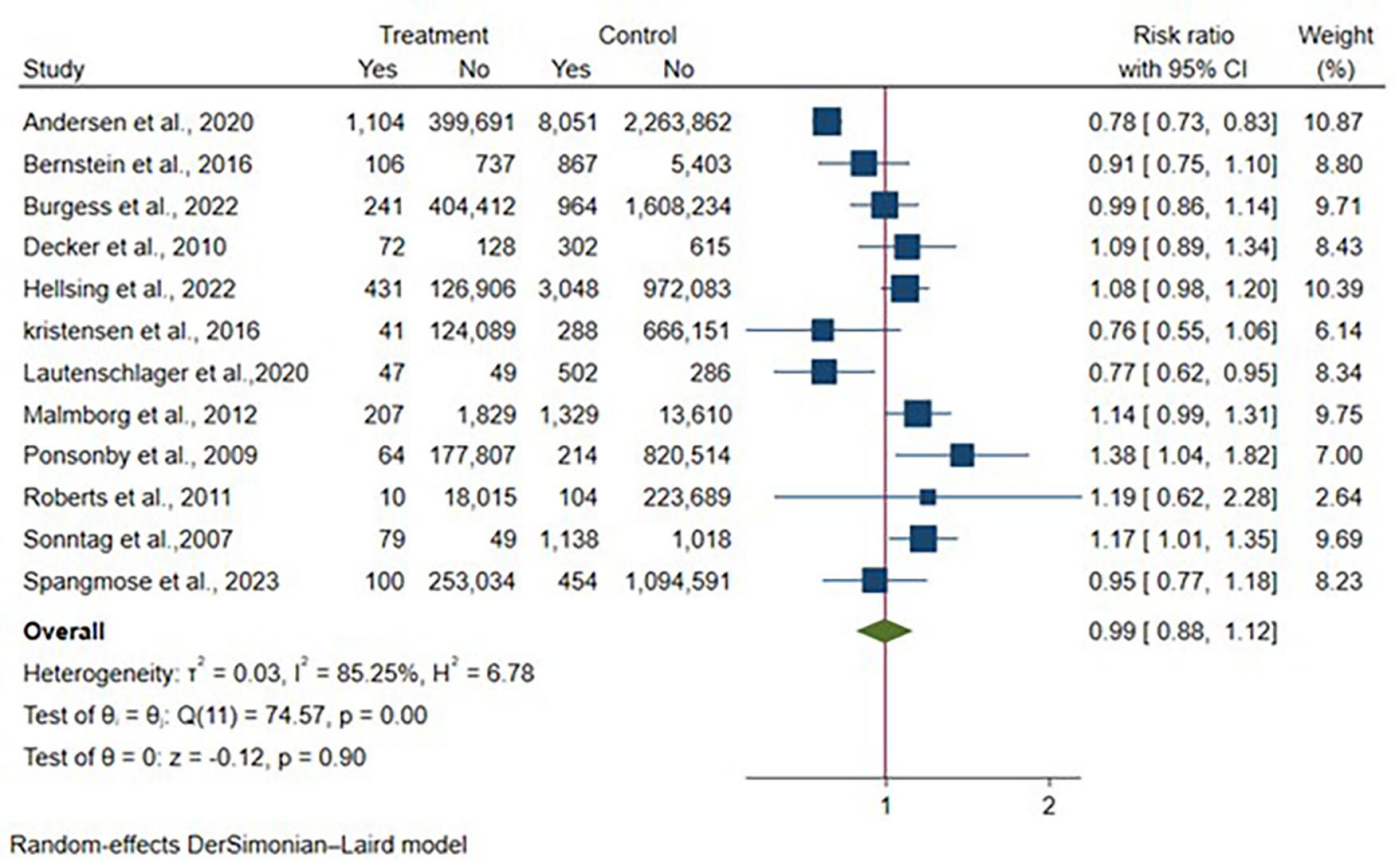
Forest plot of unadjusted risk ratios for Crohn's disease development comparing cesarean section vs. vaginal delivery. Treatment group represents cesarean section deliveries; Control group represents vaginal deliveries. Risk ratio/hazard ratio/odds ratio < 1.0 favors CS (lower IBD/CD/UC risk), while values > 1.0 favor VD (lower risk). Each horizontal line represents one study's 95% confidence interval (CI), with the point estimate shown by a square marker (size proportional to study weight). The vertical line at 1.0 represents no association. The diamond represents the pooled effect estimate and 95% CI across all studies. I^2^, tau^2^, H^2^, and Cochran's *Q* test assess between-study heterogeneity (see Methods for interpretation).

Twelve articles provided Crohn's disease (CD) case counts, representing 7,693,604 vaginal deliveries and 1,510,002 cesarean deliveries. The meta-analysis demonstrated no significant association between delivery mode and CD development [RR: 0.99, 95% CI: (0.88–1.12), *p* = 0.90] (Figure 3). Considerable heterogeneity was present across studies (I^2^ = 85.25%, *p* < 0.01).

#### 3.4.3 Ulcerative colitis

**Figure 4:**
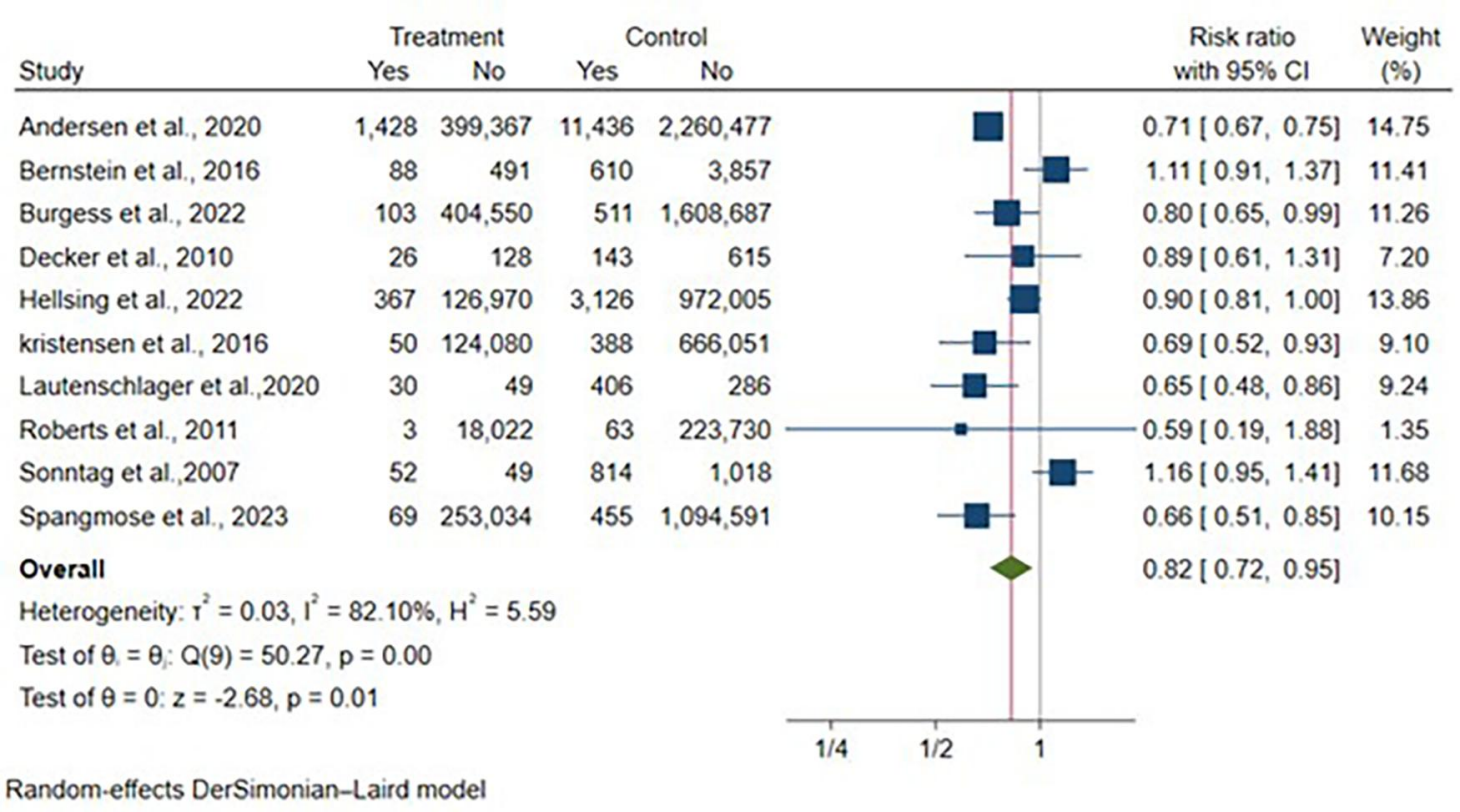
Forest plot of unadjusted risk ratios for ulcerative colitis development comparing cesarean section vs. vaginal delivery. Treatment group represents cesarean section deliveries; Control group represents vaginal deliveries. Risk ratio/hazard ratio/odds ratio <1.0 favors CS (lower IBD/CD/UC risk), while values >1.0 favor VD (lower risk). Each horizontal line represents one study's 95% confidence interval (CI), with the point estimate shown by a square marker (size proportional to study weight). The vertical line at 1.0 represents no association. The diamond represents the pooled effect estimate and 95% CI across all studies. I^2^, tau^2^, H^2^, and Cochran's *Q* test assess between-study heterogeneity (see Methods for interpretation).

Ten articles reported UC case counts, encompassing 6,857,937 vaginal deliveries and 1,330,095 cesarean deliveries. Cesarean section was associated with a statistically significant 18% reduction in UC development risk compared to vaginal delivery [RR: 0.82, 95% CI: (0.72–0.95), *p* = 0.01**]** (Figure 4). Substantial heterogeneity was observed among included studies (I^2^ = 82.1%, *p* < 0.01).

### 3.5 Meta-Analysis of adjusted hazard ratios

#### 3.5.1 Inflammatory bowel disease

**Figure 5:**
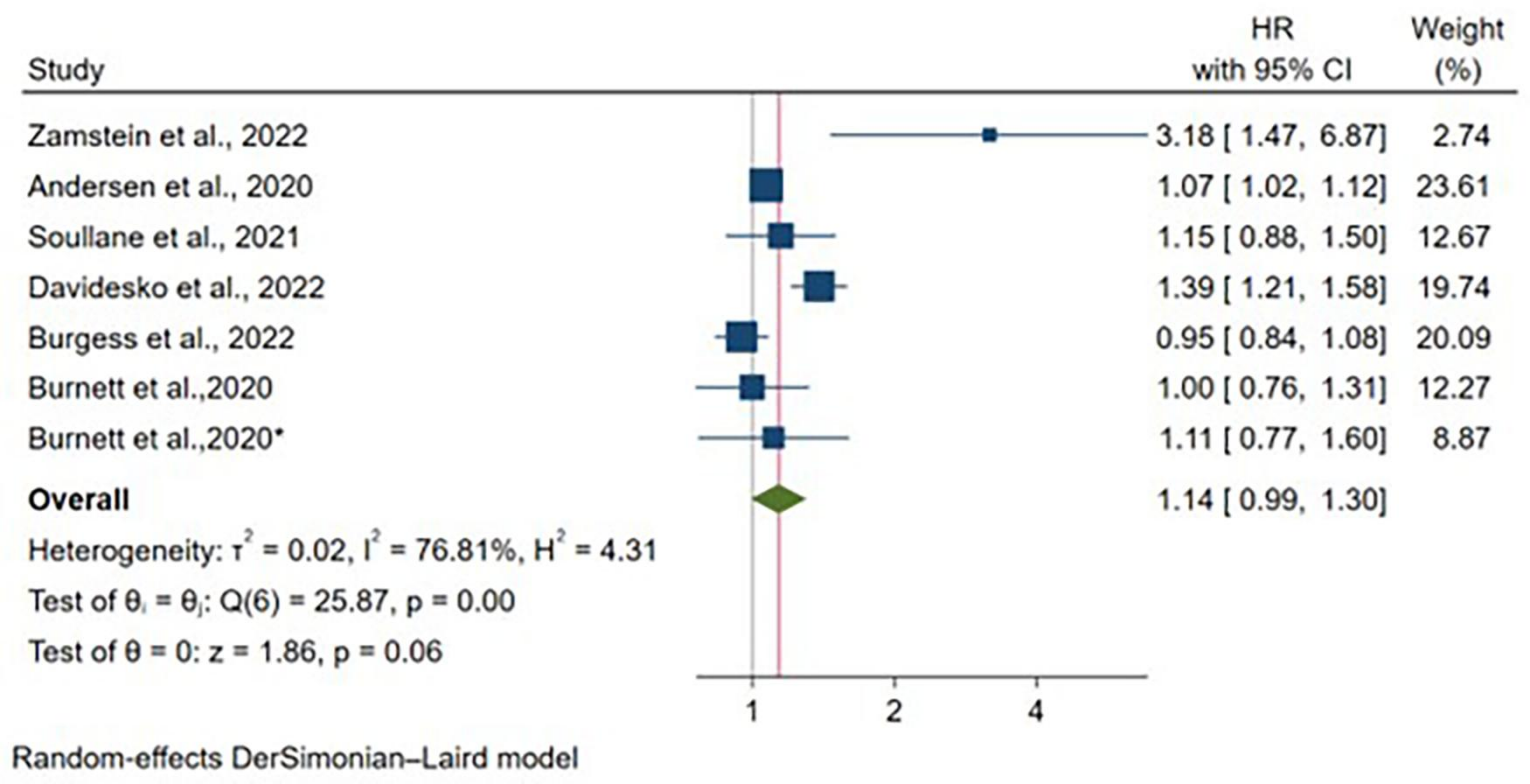
Forest plot of adjusted hazard ratios for inflammatory bowel disease development comparing cesarean section vs. vaginal delivery. Treatment group represents cesarean section deliveries; Control group represents vaginal deliveries. Risk ratio/hazard ratio/odds ratio <1.0 favors CS (lower IBD/CD/UC risk), while values >1.0 favor VD (lower risk). Each horizontal line represents one study's 95% confidence interval (CI), with the point estimate shown by a square marker (size proportional to study weight). The vertical line at 1.0 represents no association. The diamond represents the pooled effect estimate and 95% CI across all studies. I^2^, tau^2^, H^2^, and Cochran's *Q* test assess between-study heterogeneity (see Methods for interpretation).

Six articles reported adjusted hazard ratio estimates for IBD, comprising 4,930,179 vaginal deliveries and 1,143,433 cesarean deliveries. Following adjustment for maternal age and various study-specific covariates, no significant association was detected between delivery mode and IBD risk [HR: 1.14, 95% CI: (0.99–1.30), *p* = 0.06] (Figure 5). Moderate heterogeneity persisted across studies (I^2^ = 76.81%, *p* < 0.01). All studies incorporated maternal age as a covariate, while adjustment for additional variables varied across individual studies (Supplementary Table S3). Notably, Burnett et al., 2020 (22) contributed two distinct cohorts (clinical and administrative) with separately reported estimates.

#### 3.5.2 Crohn's disease

**Figure 6:**
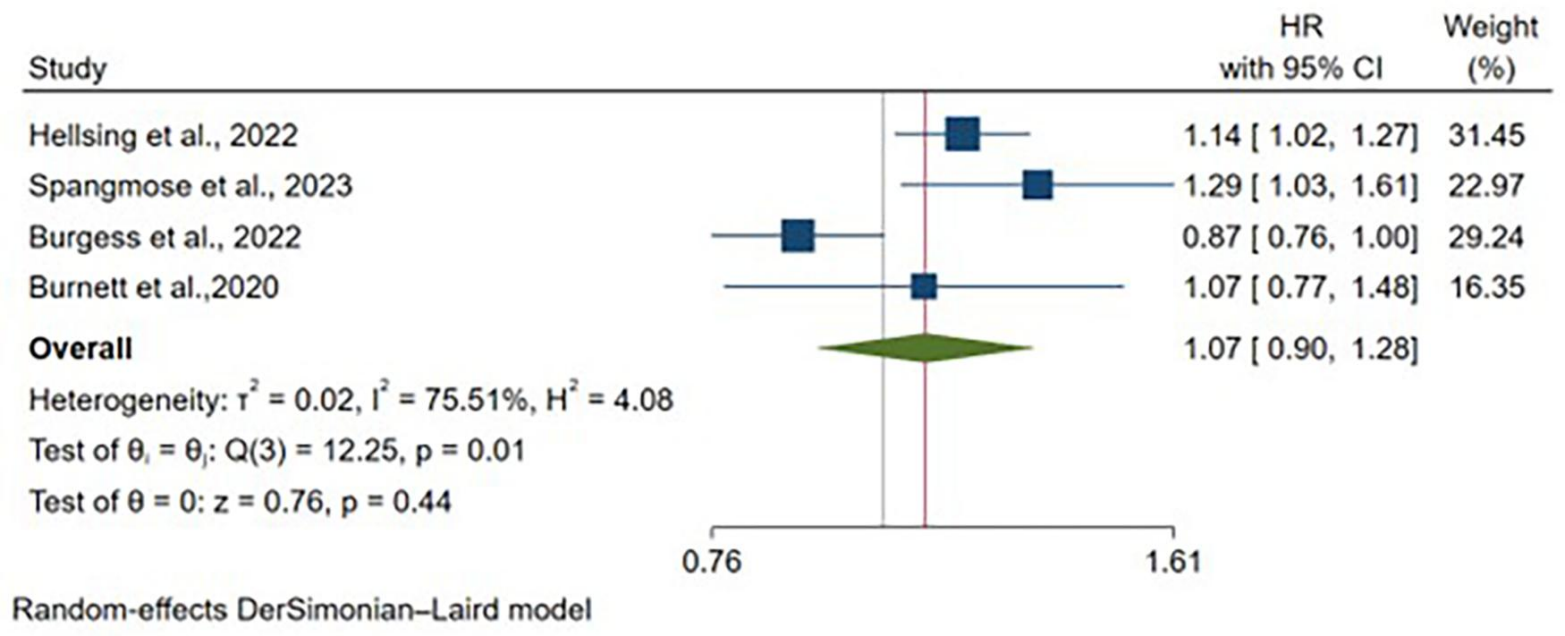
Forest plot of adjusted hazard ratios for crohn's disease development comparing cesarean section vs. vaginal delivery. Treatment group represents cesarean section deliveries; Control group represents vaginal deliveries. Risk ratio/hazard ratio/odds ratio <1.0 favors CS (lower IBD/CD/UC risk), while values >1.0 favor VD (lower risk). Each horizontal line represents one study's 95% confidence interval (CI), with the point estimate shown by a square marker (size proportional to study weight). The vertical line at 1.0 represents no association. The diamond represents the pooled effect estimate and 95% CI across all studies. I^2^, tau^2^, H^2^, and Cochran's *Q* test assess between-study heterogeneity (see Methods for interpretation).

Four articles provided adjusted hazard ratio estimates for CD, representing 3,881,286 vaginal deliveries and 846,441 cesarean deliveries. After controlling for maternal age and study-specific confounders, no significant association emerged between delivery mode and CD risk [HR: 1.07, 95% CI: (0.90–1.28), *p* = 0.44] (Figure 6). Moderate heterogeneity was evident (I^2^ = 75.51%, *p* = 0.01). Maternal age adjustment was universal across studies, with variability in additional covariate adjustment (Supplementary Table S3)**.**

#### 3.5.3 Ulcerative colitis

**Figure 7:**
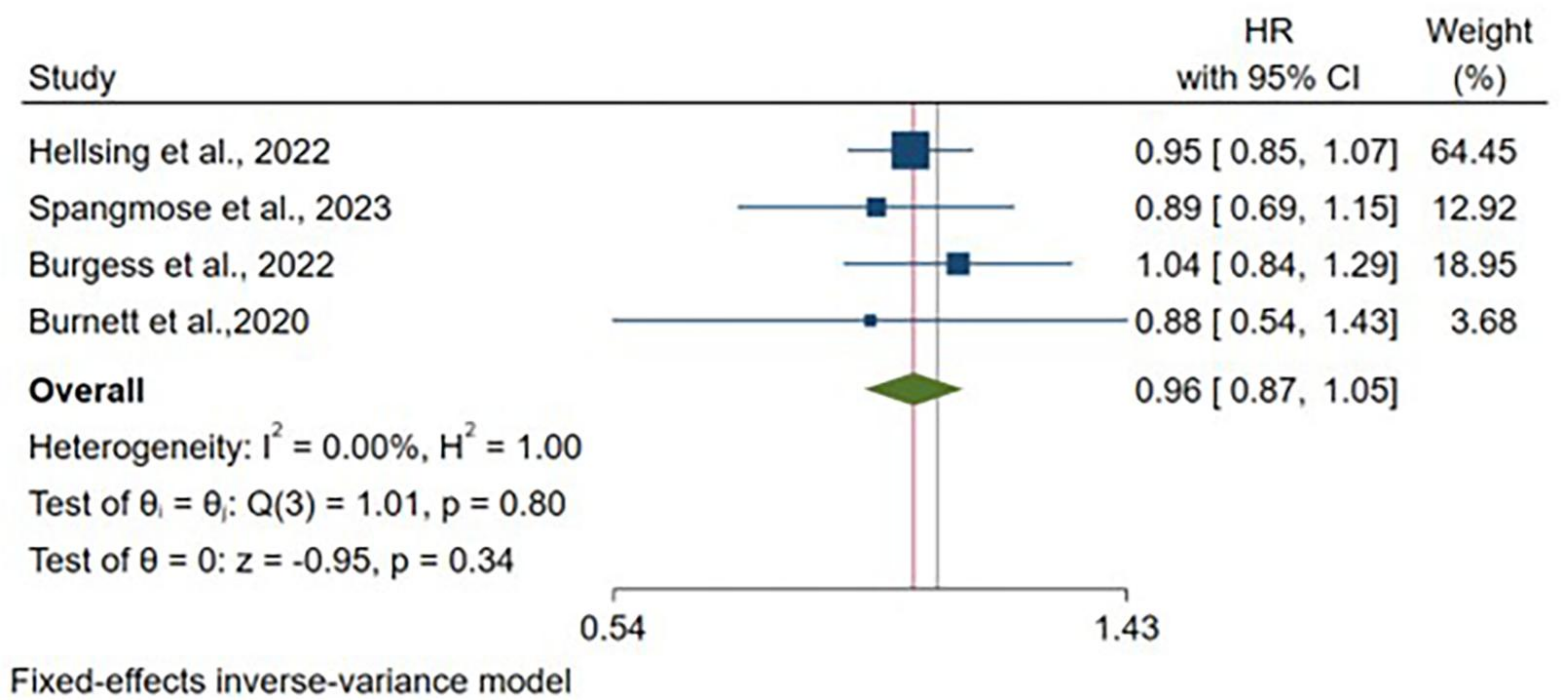
Forest plot of adjusted hazard ratios for ulcerative colitis development comparing cesarean section vs. vaginal delivery. Treatment group represents cesarean section deliveries; Control group represents vaginal deliveries. Risk ratio/hazard ratio/odds ratio <1.0 favors CS (lower IBD/CD/UC risk), while values >1.0 favor VD (lower risk). Each horizontal line represents one study's 95% confidence interval (CI), with the point estimate shown by a square marker (size proportional to study weight). The vertical line at 1.0 represents no association. The diamond represents the pooled effect estimate and 95% CI across all studies. I^2^, tau^2^, H^2^, and Cochran's *Q* test assess between-study heterogeneity (see Methods for interpretation).

Four articles reported adjusted hazard ratio estimates for UC, encompassing 3,881,286 vaginal deliveries and 846,441 cesarean deliveries. Following adjustment for maternal age and study-specific covariates, no significant association was identified between delivery mode and UC risk [HR: 0.96, 95% CI: (0.87–1.05), *p* = 0.34] (Figure 7). All included studies adjusted for maternal age, with variation in additional covariate selection across studies (Supplementary Table S3).

### 3.6 Meta-Analysis of adjusted odds ratios

#### 3.6.1 Inflammatory bowel disease

**Figure 8:**
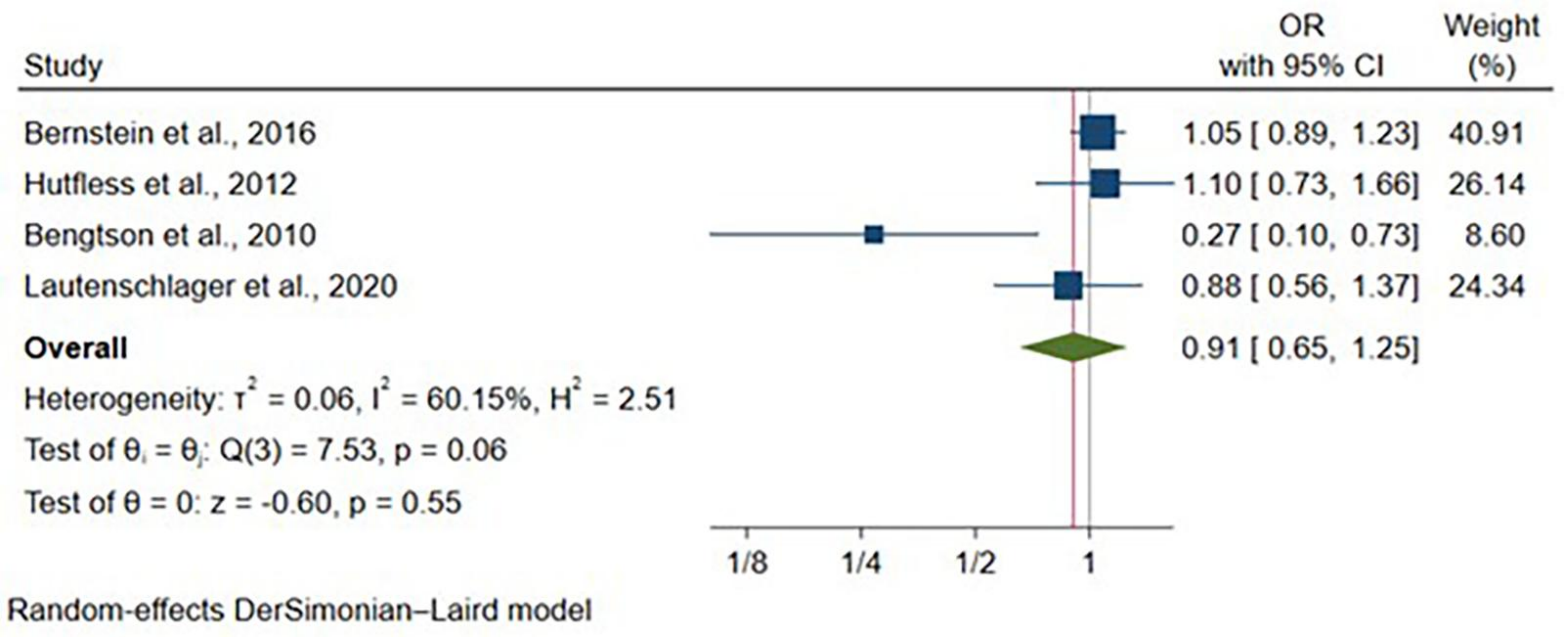
Forest plot of adjusted odds ratios for inflammatory bowel disease development comparing cesarean section vs. vaginal delivery. Treatment group represents cesarean section deliveries; Control group represents vaginal deliveries. Risk ratio/hazard ratio/odds ratio < 1.0 favors CS (lower IBD/CD/UC risk), while values > 1.0 favor VD (lower risk). Each horizontal line represents one study's 95% confidence interval (CI), with the point estimate shown by a square marker (size proportional to study weight). The vertical line at 1.0 represents no association. The diamond represents the pooled effect estimate and 95% CI across all studies. I^2^, tau^2^, H^2^, and Cochran's *Q* test assess between-study heterogeneity (see Methods for interpretation).

Four articles reported adjusted odds ratio estimates for IBD, including 656,638 vaginal deliveries and 58,733 cesarean deliveries. After adjustment for child's sex and study-specific covariates, no significant association was observed between delivery mode and IBD odds [OR: 0.91, 95% CI: (0.65–1.25), *p* = 0.55] (Figure 8). Moderate heterogeneity was present (I^2^ = 60.15%, *p* = 0.06). Child's sex was included as a covariate in all studies, with variable adjustment for additional factors (Supplementary Table S3).

#### 3.6.2 Crohn's disease

**Figure 9:**
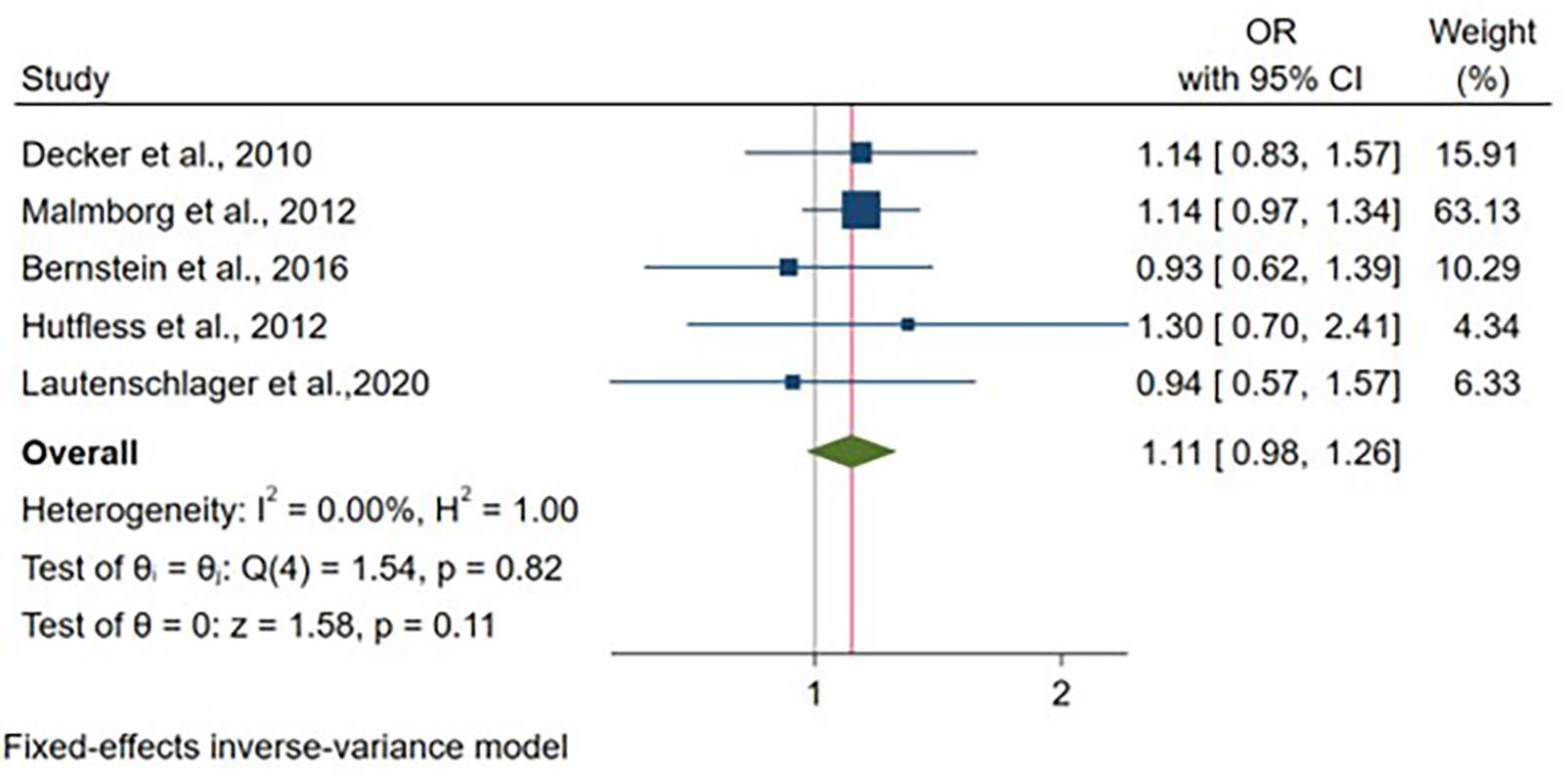
Forest plot of adjusted odds ratios for crohn's disease development comparing cesarean section vs. vaginal delivery. Treatment group represents cesarean section deliveries; Control group represents vaginal deliveries. Risk ratio/hazard ratio/odds ratio <1.0 favors CS (lower IBD/CD/UC risk), while values >1.0 favor VD (lower risk). Each horizontal line represents one study's 95% confidence interval (CI), with the point estimate shown by a square marker (size proportional to study weight). The vertical line at 1.0 represents no association. The diamond represents the pooled effect estimate and 95% CI across all studies. I^2^, tau^2^, H^2^, and Cochran's *Q* test assess between-study heterogeneity (see Methods for interpretation).

Five articles provided adjusted odds ratio estimates for CD, representing 30,593 vaginal deliveries and 4,416 cesarean deliveries. No significant association was detected between delivery mode and CD odds after controlling for child's sex and study-specific covariates [OR: 1.11, 95% CI: (0.98–1.26), *p* = 0.11] (Figure 9). Notably, these studies demonstrated homogeneity (I^2^ = 0%, *p* = 0.82). All studies adjusted for child's sex, with heterogeneity in additional covariate selection (Supplementary Table S3).

#### 3.6.3 Ulcerative colitis

**Figure 10:**
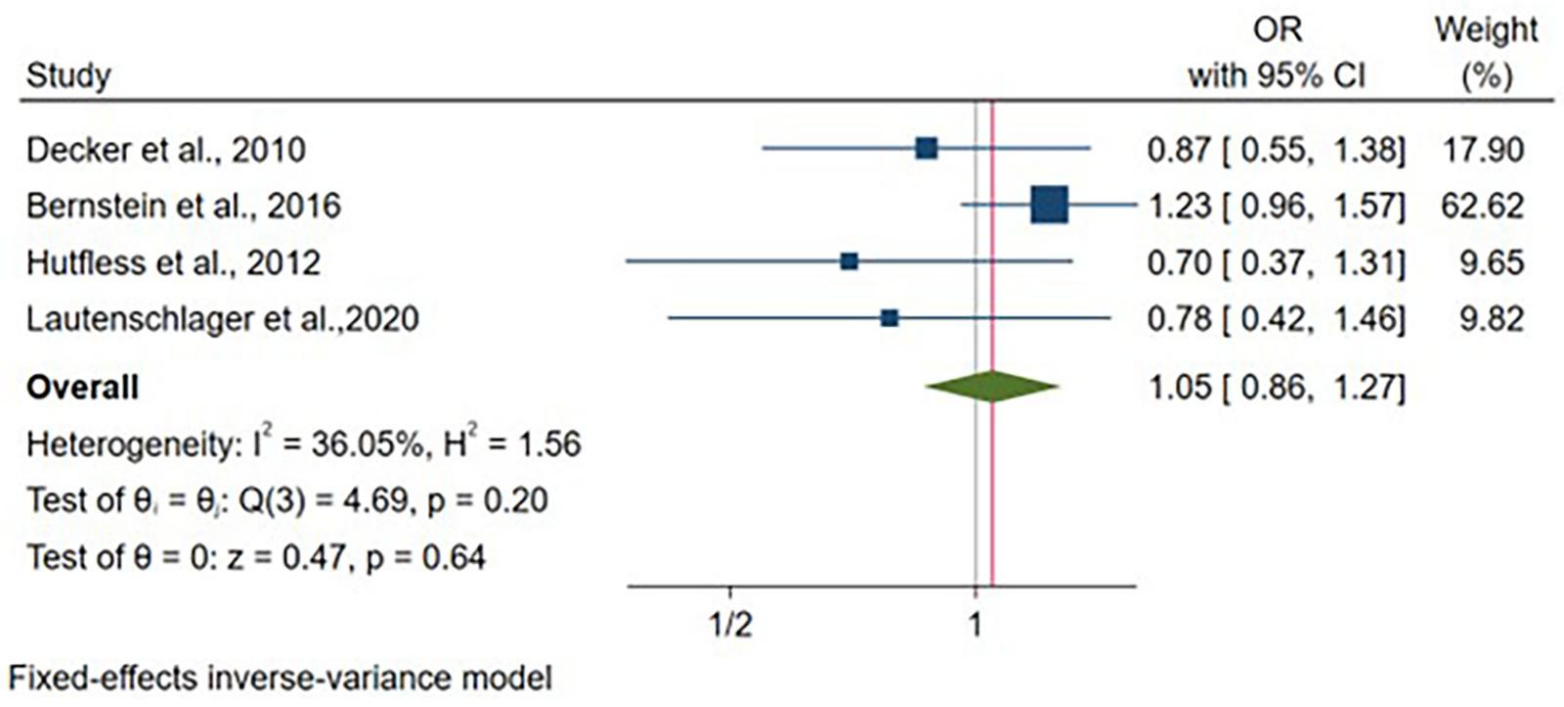
Forest plot of adjusted odds ratios for ulcerative colitis development comparing cesarean section vs. vaginal delivery. Treatment group represents cesarean section deliveries; Control group represents vaginal deliveries. Risk ratio/hazard ratio/odds ratio < 1.0 favors CS (lower IBD/CD/UC risk), while values > 1.0 favor VD (lower risk). Each horizontal line represents one study's 95% confidence interval (CI), with the point estimate shown by a square marker (size proportional to study weight). The vertical line at 1.0 represents no association. The diamond represents the pooled effect estimate and 95% CI across all studies. I^2^, tau^2^, H^2^, and Cochran's *Q* test assess between-study heterogeneity (see Methods for interpretation).

Four articles reported adjusted odds ratio estimates for UC, encompassing 15,654 vaginal deliveries and 2,380 cesarean deliveries. Following adjustment for child's sex and study-specific covariates, no significant association was identified between delivery mode and UC odds [OR: 1.05, 95% CI: (0.86–1.27), *p* = 0.64] (Figure 10). The included studies demonstrated homogeneity (I^2^ = 36.05%, *p* = 0.20). Child's sex adjustment was universal, with variability in additional covariates across studies (Supplementary Table S3).

### 3.7 Sensitivity analysis

A leave-one-out sensitivity analysis was performed to evaluate the robustness and stability of the pooled estimates. While the majority of effect estimates demonstrated stability across sequential study removal, the adjusted hazard ratios for both IBD and CD exhibited sensitivity to the inclusion of Burgess et al., 2022 (16), which was identified as an influential outlier in both analyses (Supplementary Figures S1–S9).

### 3.8 Publication bias assessment

**Figure 11:**
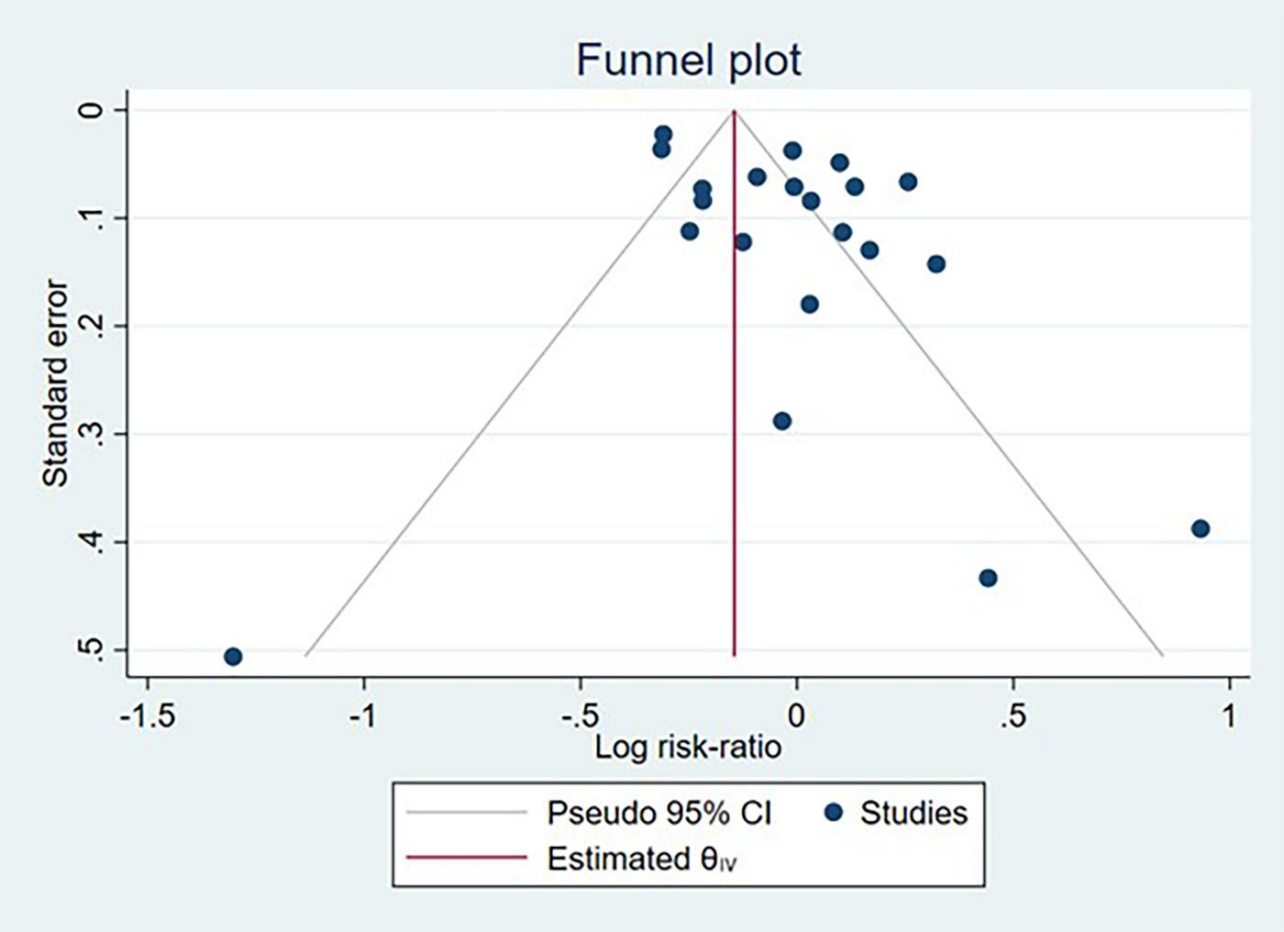
Funnel plot assessing publication bias among studies examining the association between cesarean section and inflammatory bowel disease development.

**Figure 12:**
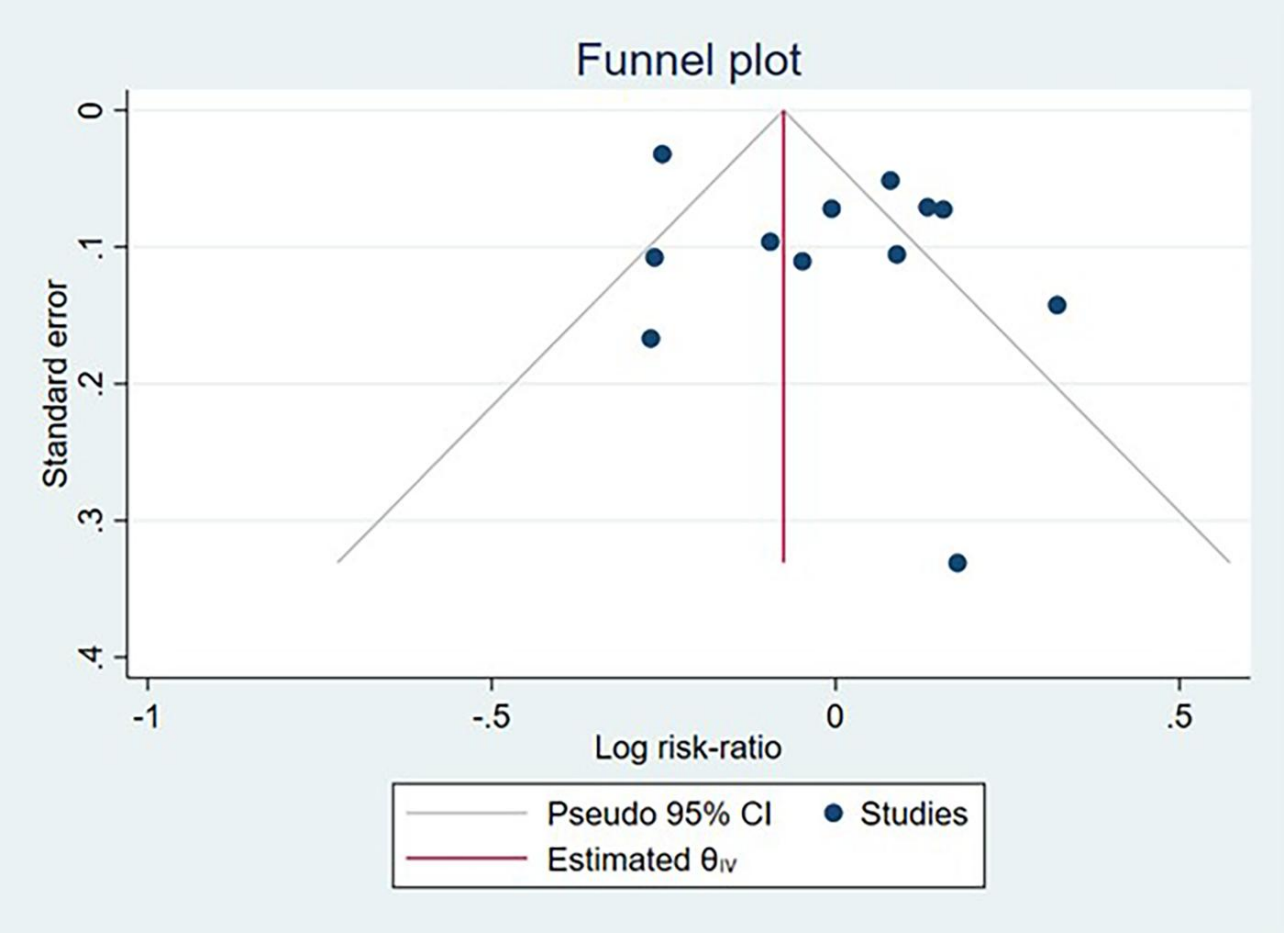
Funnel plot assessing publication bias among studies examining the association between cesarean section and crohn's disease development.

**Figure 13:**
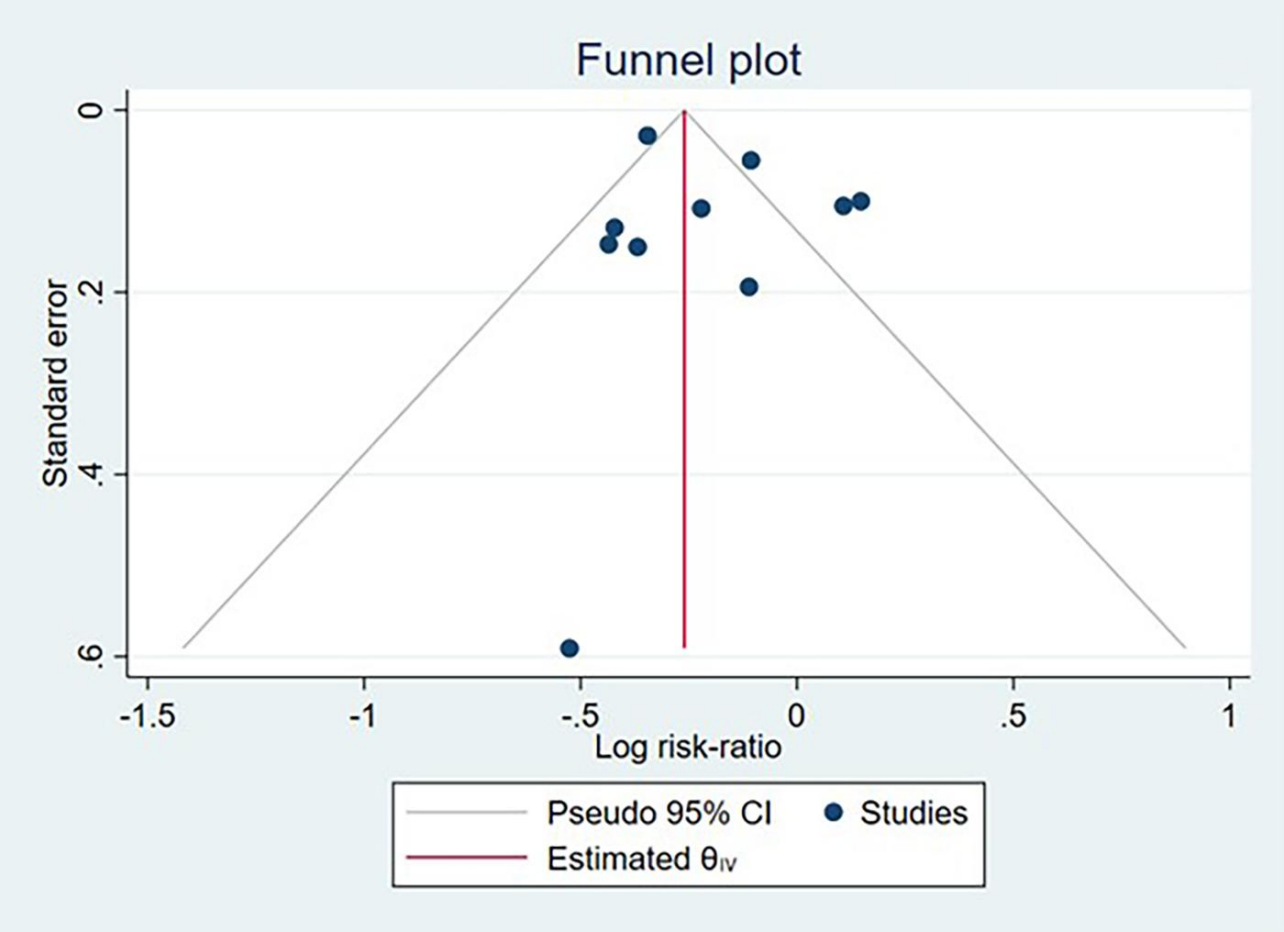
Funnel plot assessing publication bias among studies examining the association between cesarean section and ulcerative colitis development.

Potential publication bias was evaluated for studies reporting IBD, CD, and UC case counts through visual inspection of funnel plots and formal statistical testing using Egger's regression test. Funnel plots demonstrated symmetrical distribution for all three outcomes. Egger's test yielded non-significant results for IBD (*p* = 0.316), CD (*p* = 0.65), and UC (*p* = 0.573), providing no substantial evidence of publication bias across the included studies (Figures 11–13).

## 4 Discussion

This systematic review and meta-analysis of 22 observational studies encompassing more than 13 million births identified no significant association between mode of delivery and the subsequent development of IBD, CD, or UC in offspring.

The overall pooled risk ratio demonstrated no significant association between delivery mode and IBD development, although substantial heterogeneity was observed (I^2^ = 90.66%). Subgroup analysis revealed striking regional variation, with CS associated with decreased IBD risk in Denmark, Switzerland, and Norway, but increased risk in Germany and Australia. These regional differences suggest potential influences from genetic susceptibility, environmental exposures, and healthcare practice variations. These findings align with two previously published meta-analyses (19, 40) that similarly reported no overall association, though without exploring regional heterogeneity.

For Crohn's disease, the pooled risk ratio, adjusted hazard ratio, and adjusted odds ratio analyses all consistently indicated no significant association with delivery mode, consistent with Frias Gomes et al., 2021 (18). For ulcerative colitis, unadjusted analyses suggested a protective effect of CS (RR: 0.82), which was eliminated in adjusted hazard ratio (HR: 0.96) and odds ratio (OR: 1.05) analyses. This pattern strongly suggests confounding by indication rather than true biological protection. Maternal IBD status, socioeconomic factors, and pregnancy complications, all potential indications for CS, independently influence offspring IBD risk. The attenuation following adjustment for these confounders indicates the unadjusted protective signal likely represents a methodological artifact rather than genuine protection.

Despite well-established biological mechanisms linking delivery mode to microbiome development and immune programming, our null findings suggest these pathways do not translate into measurable population-level IBD risk. During vaginal delivery, neonates are exposed to maternal vaginal and intestinal microbiota, while cesarean-delivered infants are colonized by skin-associated or environmental microbes, with these differences persisting during critical immune development periods (42). Disrupted early colonization can reduce immune-regulating metabolites and alter T-regulatory and innate immune cell signaling, mechanisms strongly implicated in IBD pathogenesis (43). Furthermore, peripartum antibiotic exposure, more common after CS, can further disrupt microbial succession, with several studies reporting elevated childhood IBD rates following early-life antibiotic use (44, 45).

However, several factors explain why delivery mode shows no measurable epidemiological effect. First, postnatal factors, including breastfeeding, antibiotic exposure, diet, and environmental microbial diversity, likely exert stronger influences on microbiome development than initial delivery mode, effectively overshadowing transient early differences. Second, the infant microbiome demonstrates remarkable resilience, with compositional differences typically converging by 12–24 months. Third, IBD pathogenesis involves complex genetic-environmental interactions, with delivery mode representing only a minor factor whose effect may be too small to detect against more influential risk determinants. The observed divergence between CD and UC, though primarily in unadjusted analyses, may reflect distinct pathogenic mechanisms, as UC represents mucosal colonic inflammation while CD involves transmural inflammation with broader genetic-environmental interplay (46, 47). This emphasizes the importance of examining these subtypes separately in future research.

Our findings align partially with existing literature while revealing important complexities. Several high-quality meta-analyses and registry-based cohorts have reported no consistent association, supporting our primary findings. Conversely, some large population studies have reported modest increases in childhood IBD risk, particularly for CD, following cesarean delivery (11, 17). This heterogeneity likely reflects differences in confounder adjustment, with our finding that adjusted analyses yield null results suggesting inadequate confounder control may explain some previously reported positive associations. Additionally, populations differ in CS rates, indications, and practices, while outcome ascertainment and follow-up duration vary substantially across studies.

Despite the global rise in CS rates over recent decades (2, 41), and concerns about potential immunological effects (25), these findings provide important clinical reassurance. IBD risk should not influence delivery mode decisions, and clinicians can confidently reassure patients that medically indicated CS will not increase their child's IBD risk. Clinical efforts to prevent childhood IBD should focus on potentially more modifiable factors, including judicious early-childhood antibiotic use and breastfeeding promotion.

This investigation benefits from substantial pooled sample size, broad geographical representation, and rigorous analytical approaches. However, the observational nature of included studies introduces potential residual confounding. Critical early-life exposures including breastfeeding, antibiotic timing, and environmental factors were inconsistently documented, limiting exploration of effect modifiers. Few studies distinguished elective from emergency CS or adjusted for CS indication, and maternal IBD status was inconsistently reported. Regional subgroup analyses were constrained by limited studies from certain areas, and substantial heterogeneity (I^2^ > 80%) suggests important unmeasured differences between studies. Furthermore, the absence of individual-level genetic data and microbiome profiling precluded direct evaluation of genetic susceptibility and microbiome-mediated pathways. Future research should employ prospective designs with comprehensive early-life exposure assessment, integrate microbiome and genomic data, examine gene-environment interactions, and include more diverse cohorts to clarify whether specific populations show heightened susceptibility to early-life microbial disruption.

## 5 Conclusion

This comprehensive systematic review and meta-analysis of over 13 million births identified no significant association between cesarean section and the risk of IBD, including CD and UC. While unadjusted analyses suggested modest CS protection for UC, this disappeared after confounder adjustment, indicating confounding by indication rather than true protection. These findings demonstrate that delivery mode does not independently influence IBD development, with genetic predisposition and postnatal environmental exposures likely playing more substantial roles. These results provide clinical reassurance that medically indicated cesarean section does not confer increased long-term IBD risk in offspring.

## Data Availability

The original contributions presented in the study are included in the article/Supplementary Material, further inquiries can be directed to the corresponding author/s.
