# Supplementary material for: Mode of birth and risk of inflammatory bowel disease in offspring: an updated systematic review and meta-analysis
Source: Front Reprod Health. 2026 Apr 8;8:1776110. doi: 10.3389/frph.2026.1776110 (PMC13099911; doi:10.3389/frph.2026.1776110)
Supplement: Supplementary file 1 [file Table1.docx]

**Table S1: Search strategy**

| **Database** | **Search Strategy** |
| --- | --- |
| **PubMed** | **(("Cesarean Section"[Mesh] OR "Cesarean Section"[tiab] OR "Caesarean Section"[tiab] OR "C-section"[tiab] OR "Cesarean delivery"[tiab] OR "Caesarean delivery"[tiab] OR "surgical delivery"[tiab] OR "operative delivery"[tiab]) AND ("Inflammatory Bowel Diseases"[Mesh] OR "inflammatory bowel disease"[tiab] OR "inflammatory bowel diseases"[tiab] OR IBD[tiab] OR "Crohn Disease"[Mesh] OR "Crohn's disease"[tiab] OR "Crohns disease"[tiab] OR "Ulcerative Colitis"[Mesh] OR "ulcerative colitis"[tiab]))** |
| **Web of Science (Core Collection)** | **TS=(("cesarean section" OR "caesarean section" OR "c-section" OR "cesarean delivery" OR "caesarean delivery" OR "surgical delivery" OR "operative delivery") AND ("inflammatory bowel disease" OR "inflammatory bowel diseases" OR IBD OR "Crohn's disease" OR "Crohns disease" OR "ulcerative colitis"))** |
| **Scopus** | **TITLE-ABS-KEY(("cesarean section" OR "caesarean section" OR "c-section" OR "cesarean delivery" OR "caesarean delivery" OR "surgical delivery" OR "operative delivery") AND ("inflammatory bowel disease" OR "inflammatory bowel diseases" OR IBD OR "Crohn's disease" OR "Crohns disease" OR "ulcerative colitis"))** |
| **Cochrane CENTRAL (Cochrane Library)** | **("cesarean section" OR "caesarean section" OR "c-section" OR "cesarean delivery" OR "caesarean delivery" OR "surgical delivery" OR "operative delivery") AND ("inflammatory bowel disease" OR "inflammatory bowel diseases" OR IBD OR "Crohn's disease" OR "Crohns disease" OR "ulcerative colitis")** |

**Table S2: Excluded studies through full-text screening**

| Study title | Study ID | Reason for exclusion |
| --- | --- | --- |
| Fecal Calprotectin Level in Term and Preterm Babies before and after Starting of Feeding | Romih et al., 2021 | Did not assess CS-IBD association nor reported IBD, CD, or UC counts or effect sized |
| Faecal calprotectin levels during the first year of life in healthy children | Şahin et al., 2020 | Did not assess CS-IBD association nor reported IBD, CD, or UC counts or effect sized and is a cross-sectional study |
| Clinical and epidemiological features of ulcerative colitis in children of the Rostov region | Eliseeva et al., 2024 | Full-text was not available in English |
| Early risk factors for the development of inflammatory bowel disease in the pediatric population of the Kuyavian-Pomeranian Voivodeship | Dolińska et al., 2018 | Full-text was not available in English |
| Delivery and feeding mode affects fecal calprotectin levels in infants <7months old | Lee et al., 2017 | Did not assess CS-IBD association nor reported IBD, CD, or UC counts or effect sized |
| Mode of delivery—impact on risk of noncommunicable diseases | Miettinen et al., 2015 | An editorial letter |
| Risk Factors for Developing Inflammatory Bowel Disease Within and Across Families with a Family History of IBD | Torres et al., 2023 | Studied the risk factors for IBD within and across nuclear families with a first degree relative diagnosed with IBD |
| Cesarean section and chronic immune disorders | Sevelsted et al., 2015 | An editorial comment |
|  |  |  |
| Early Life and Childhood Environmental Exposures, More Than Genetic Predisposition, Influence Age of Diagnosis in a Diverse Cohort of 2952 Patients With IBD | Khakoo et al., 2024 | Did not include a mode of delivery comparison with a healthy control group against IBD patients |
| Analysis of Faecal Zonulin and Calprotectin Concentrations in Healthy Children During the First Two Years of Life. An Observational Prospective Cohort Study | Łoniewska et al., 2020 | Did not assess CS-IBD association nor reported IBD, CD, or UC counts or effect sized |

**Table S3: Covariates adjusted in each included study that reported an adjusted OR, HR for IBD, CD and UC risk**

| Study | Outcome | Effect Type | Adjusted Variables |
| --- | --- | --- | --- |
| Zamstein et al., 2022 | IBD | HR | Gestational age, Maternal age, Parity order, Maternal ethnicity, Gestational Diabetes Mellitus, Level of prenatal care, Gender, Birth weight |
| Andersen et al., 2020 | IBD | HR | Decade of birth, Child’s sex, Mother’s and father’s age, Mother’s diabetes, Father’s arthritis, Coeliac disease |
| Soullane et al., 2021 | IBD | HR | Maternal age, Parity, Maternal autoimmune disease, Pregnancy-related morbidity, Child sex, Socioeconomic deprivation, Time period |
| Decker et al., 2010 | CD and UC | OR | Child’s age and gender, Mode of delivery, Postnatal complications, Breastfeeding |
| Malmborg et al., 2012 | CD | OR | Maternal infection, Socioeconomic status, Delivery unit, Week of birth, Sex |
| Bernstein et al., 2016 | IBD,CD, UC | OR | Age, Sex, Area of residence |
| Hellsing et al., 2022 | CD and UC | HR | Birthweight, Congenital malformations, Maternal BMI, Maternal smoking, Parental education, Perinatal illness, Small of Gestational Age (SGA) |
| Davidesko et al., 2022 | IBD | HR | Maternal age, Ethnicity, Preterm delivery, SGA, Maternal obesity, Fetal sex, Low apgar score at 5 min (<7) |
| Spangmose et al., 2023 | CD and UC | HR | Maternal age, Maternal education, Parity, Mode of conception, Maternal preconception Immune-Mediated Inflammatory Disease (IMID), Mode of delivery, Plurality, Child’s sex, Child’s birth season |
| Burgess et al., 2022 | IBD, UC, CD | HR | Sex, Maternal age, Postcode, Deprivation, Year of birth |
| Hutfless et al., 2012 | IBD, UC, CD | OR | Race, Sex, Gestational age, Birth weight, Maternal age, Maternal hypertension, Placental/amniotic problems, Maternal infection, Maternal IBD |
| Bengtson et al., 2010 | IBD | OR | Gestational age and sex |
| Burnett et al., 2020 | IBD, UC, CD | HR | Maternal pre-pregnancy weight status, Maternal age at birth, Parity |
| Lautenschlager et al., 2020 | IBD, UC, CD | OR | Age and sex |

**Leave-one-out analysis**

**Figure S1: IBD**

**
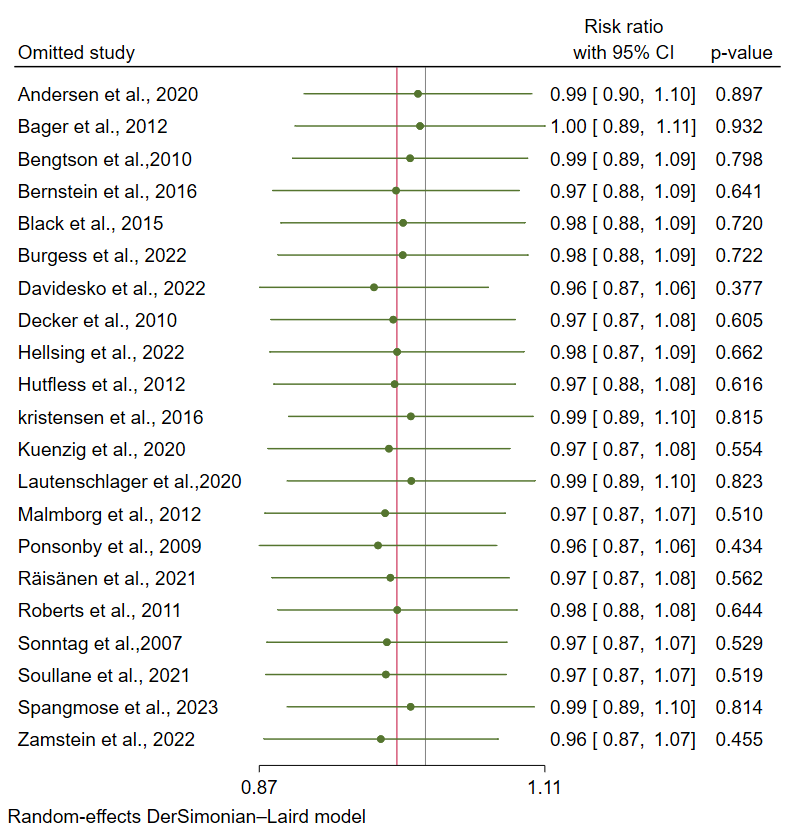
**

**Figure S2: CD**

**
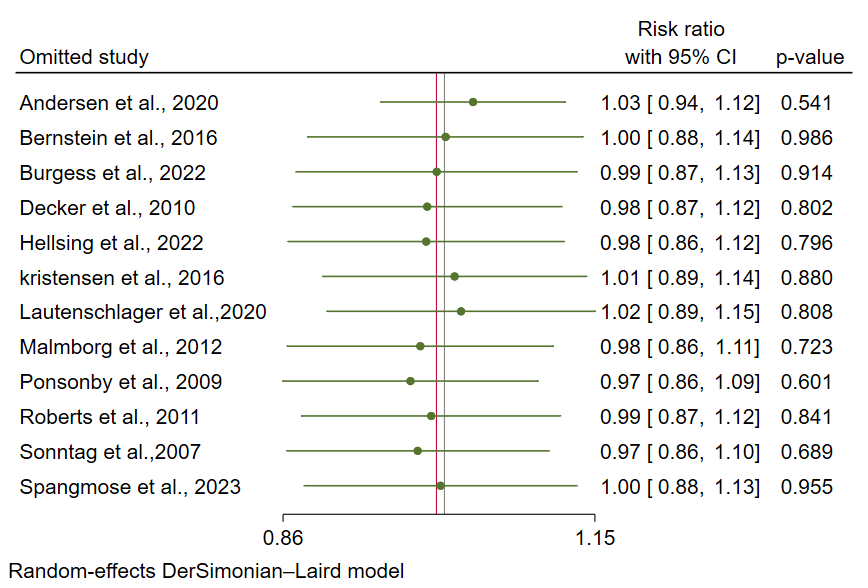
**

**Figure S3: UC**

**
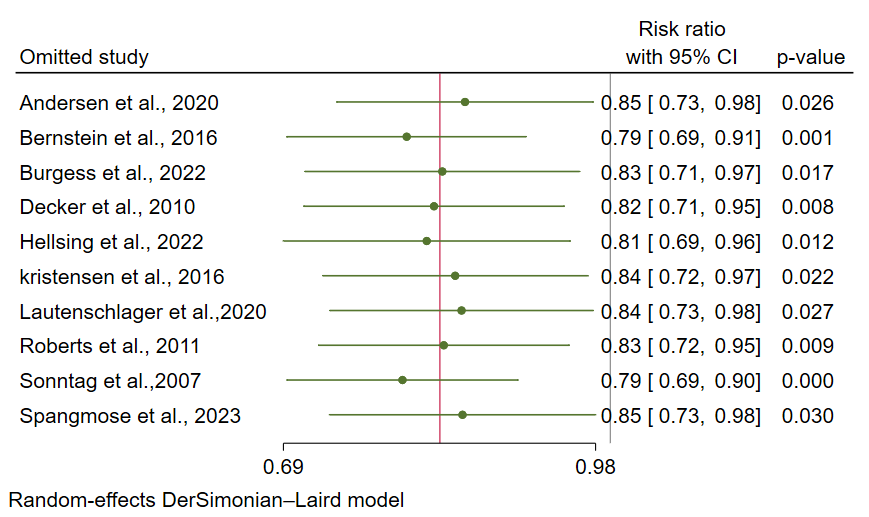
**

**Figure S4: IBD adjusted HR
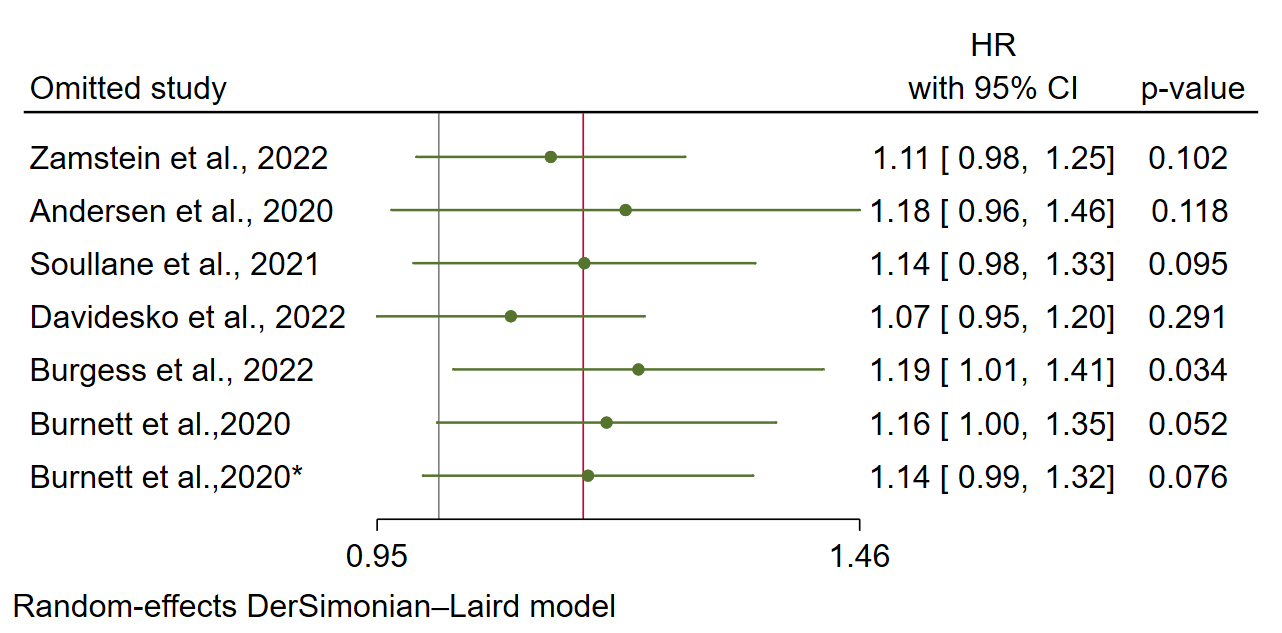
**

Burnett et al., 2020 (clinical cohort), Burnett et al., 2020* (administrative cohort)

**Figure S5: CD adjusted HR**

**
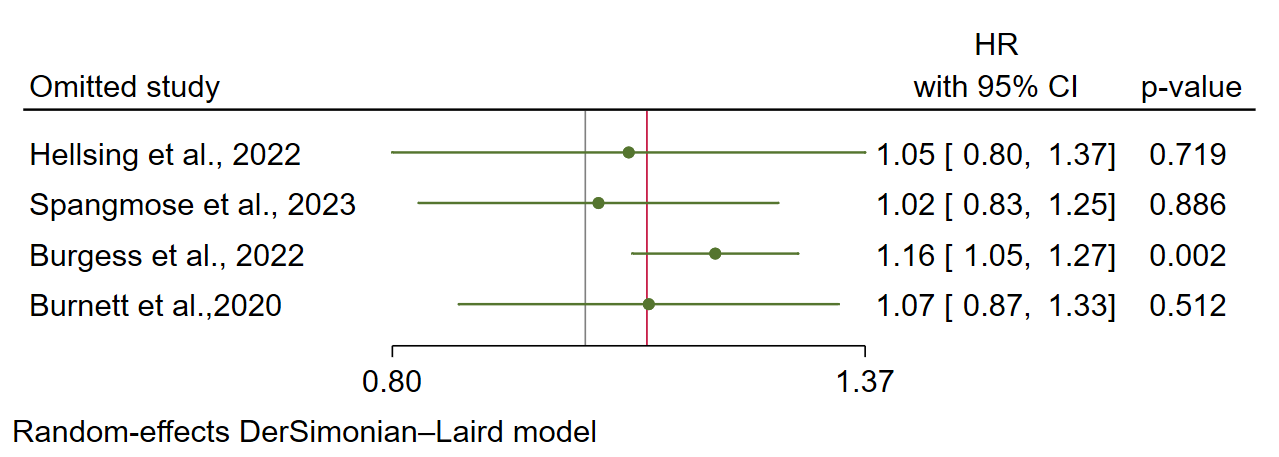
**

Burnett et al., 2020 (clinical cohort)

**Figure S6: UC adjusted HR**

**
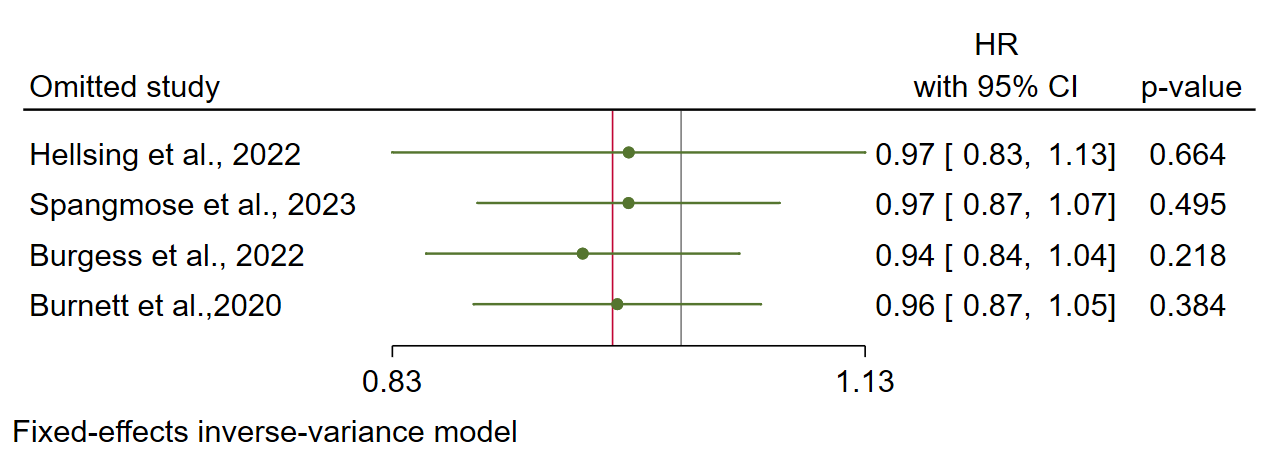
**

Burnett et al., 2020 (clinical cohort)

**Figure S7: IBD adjusted OR**

**
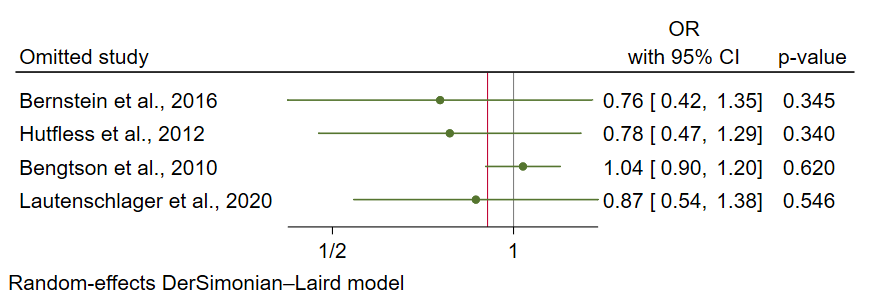
**

**Figure S8: CD adjusted OR
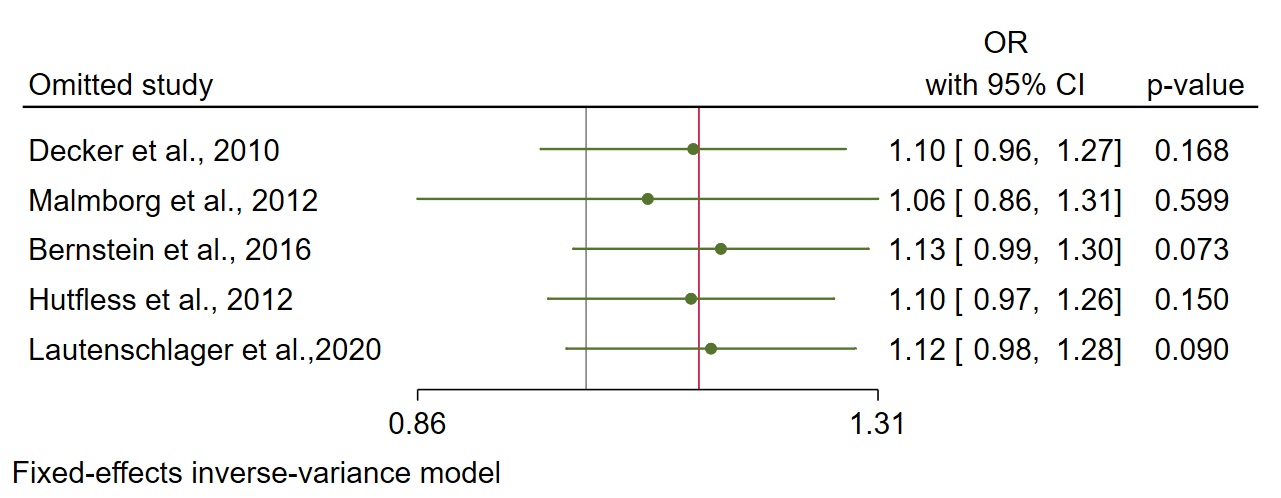
**

**Figure S9: UC adjusted OR
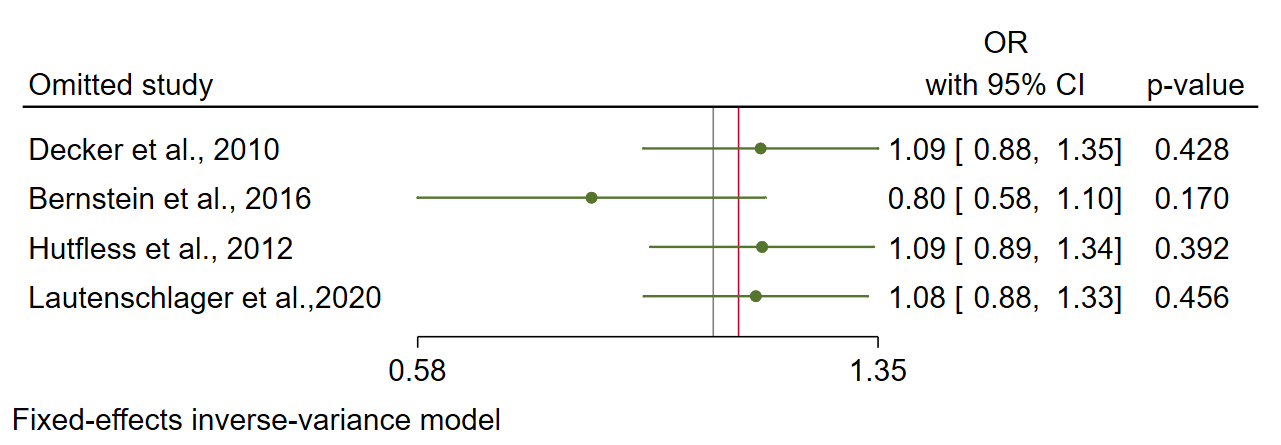
**
